# The Centrosome-Specific Phosphorylation of Cnn by Polo/Plk1 Drives Cnn Scaffold Assembly and Centrosome Maturation

**DOI:** 10.1016/j.devcel.2014.02.013

**Published:** 2014-03-31

**Authors:** Paul T. Conduit, Zhe Feng, Jennifer H. Richens, Janina Baumbach, Alan Wainman, Suruchi D. Bakshi, Jeroen Dobbelaere, Steven Johnson, Susan M. Lea, Jordan W. Raff

**Affiliations:** 1 Sir William Dunn School of Pathology, University of Oxford, South Parks Road, Oxford OX1 3RE, UK; 2 Centre for Mathematical Biology, Mathematical Institute, 24-29 St Giles, Oxford OX1 3LB, UK; 3 Max F. Perutz Laboratories, Dr. Bohr-Gasse 9, 1030 Vienna, Austria

## Abstract

Centrosomes are important cell organizers. They consist of a pair of centrioles surrounded by pericentriolar material (PCM) that expands dramatically during mitosis—a process termed centrosome maturation. How centrosomes mature remains mysterious. Here, we identify a domain in *Drosophila* Cnn that appears to be phosphorylated by Polo/Plk1 specifically at centrosomes during mitosis. The phosphorylation promotes the assembly of a Cnn scaffold around the centrioles that is in constant flux, with Cnn molecules recruited continuously around the centrioles as the scaffold spreads slowly outward. Mutations that block Cnn phosphorylation strongly inhibit scaffold assembly and centrosome maturation, whereas phosphomimicking mutations allow Cnn to multimerize in vitro and to spontaneously form cytoplasmic scaffolds in vivo that organize microtubules independently of centrosomes. We conclude that Polo/Plk1 initiates the phosphorylation-dependent assembly of a Cnn scaffold around centrioles that is essential for efficient centrosome maturation in flies.

## Highlights

• Plk1/Polo phosphorylates Cnn in vitro
• Phosphorylation appears to occur specifically at centrosomes during mitosis
• Phosphorylation allows Cnn to assemble into a dynamic scaffold around centrioles
• Cnn scaffold assembly is required for proper centrosome maturation

## Graphical Abstract

**Figure:**
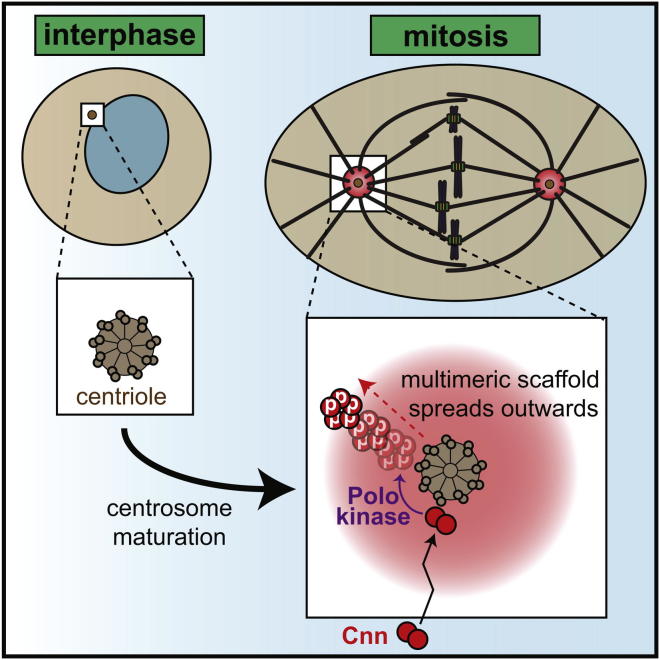

## Teaser

Centrosomes, important cellular organizers, form when centrioles recruit pericentriolar material (PCM) around themselves. Conduit et al. shed light on PCM spreading/centrosome maturation during mitosis, showing that Polo kinase initiates phosphorylation-dependent assembly of PCM protein Cnn as a scaffold around centrioles. This scaffold then spreads outward to support an expanded PCM.

## Introduction

Centrosomes are the major microtubule (MT) organizing centers in animal cells, and they influence many cell processes, including cell shape, cell polarity, and cell division (Bettencourt-Dias and Glover, 2007; Doxsey et al., 2005a). Centrosome dysfunction has been linked to many human disorders, including cancer and microcephaly (Nigg and Raff, 2009; Zyss and Gergely, 2009).

Centrosomes form when centrioles recruit a matrix of pericentriolar material (PCM) around themselves. In interphase, centrioles usually organize very little PCM, but the PCM increases dramatically during mitosis, a process termed centrosome maturation (Mahen and Venkitaraman, 2012; Mennella et al., 2013; Palazzo et al., 2000). Several hundred proteins are concentrated in the PCM, including many MT-organizing proteins, cell-cycle regulators, and cell-cycle checkpoint proteins (Alves-Cruzeiro et al., 2013; Andersen et al., 2003; Müller et al., 2010). It seems that the centrosome acts as an important regulatory center that coordinates the activity of many cytoplasmic proteins and signaling pathways (Doxsey et al., 2005b).

Several studies have pointed to the existence of a “scaffold” structure within the PCM (Dictenberg et al., 1998; Schnackenberg et al., 1998), but its molecular nature has remained elusive. Recent reports using super-resolution microscopy have revealed that a small number of centrosomal proteins are specifically oriented around the centrioles during interphase, but any organization within the expanded mitotic PCM was less apparent (Fu and Glover, 2012; Lawo et al., 2012; Mennella et al., 2012; Sonnen et al., 2012). Thus, although several proteins have been implicated in mitotic PCM assembly (Mennella et al., 2013), it remains unclear what role they play in organizing the hundreds of proteins within the PCM to form a functional mitotic centrosome.

The mitotic PCM is dynamic, because many of its proteins are continuously exchanging between their centrosomal binding sites and the cytosol. We recently showed that the conserved *Drosophila* PCM protein Centrosomin (Cnn) exhibits an unusual dynamic behavior, because its rate of exchange is much greater at the center of the PCM than at the periphery (Conduit et al., 2010). We speculated that Cnn binding sites might only be located in the center of the PCM, close to the centrioles, and that, once released from these binding sites, Cnn molecules might spread outward, forming a molecular scaffold onto which other PCM proteins might bind. This idea is attractive, because centrioles are required for efficient PCM assembly (Basto et al., 2006; Bobinnec et al., 1998; Kirkham et al., 2003), and Cnn is required for the efficient recruitment of many centrosomal proteins during mitosis (Lucas and Raff, 2007; Megraw et al., 1999, 2001). Thus, the proposed mechanism would provide a simple explanation for how centrioles might direct the assembly of an underlying scaffold that enables centrosome maturation in mitosis (Conduit and Raff, 2010). It remains unclear, however, whether Cnn molecules actually form a scaffold that spreads outward from the centrioles, how Cnn molecules assemble into such a scaffold, and how their assembly is regulated so that it occurs only around the centrioles.

Here, we use photoconversion experiments to demonstrate unambiguously that centrosomal Cnn molecules are in constant flux, incorporating into the PCM close to the centrioles and then moving slowly outward. We show that Cnn appears to be specifically phosphorylated at centrosomes and that the phosphorylation allows Cnn to assemble into a scaffold structure around the centrioles. We identify a domain within Cnn that is phosphorylated by recombinant Polo/Plk1 in vitro and contains ten potential phosphorylation sites; mutating various combinations of these sites strongly inhibits the assembly of the Cnn scaffold and centrosome maturation. Strikingly, mutating all ten of the sites to phosphomimicking residues allows the domain to efficiently assemble into stable multimers in vitro, and Cnn to spontaneously form scaffolds in vivo that can organize MTs in the absence of centrosomes. We conclude that the Polo/Plk1-dependent phosphorylation of Cnn at centrosomes promotes the assembly of a Cnn scaffold around the centrioles that spreads slowly outward to enable the dramatic expansion of the PCM during centrosome maturation in flies.

## Results

### Cnn Molecules Incorporate Only into the Center of the PCM and Then Move Slowly Outward

**Figure 1:**
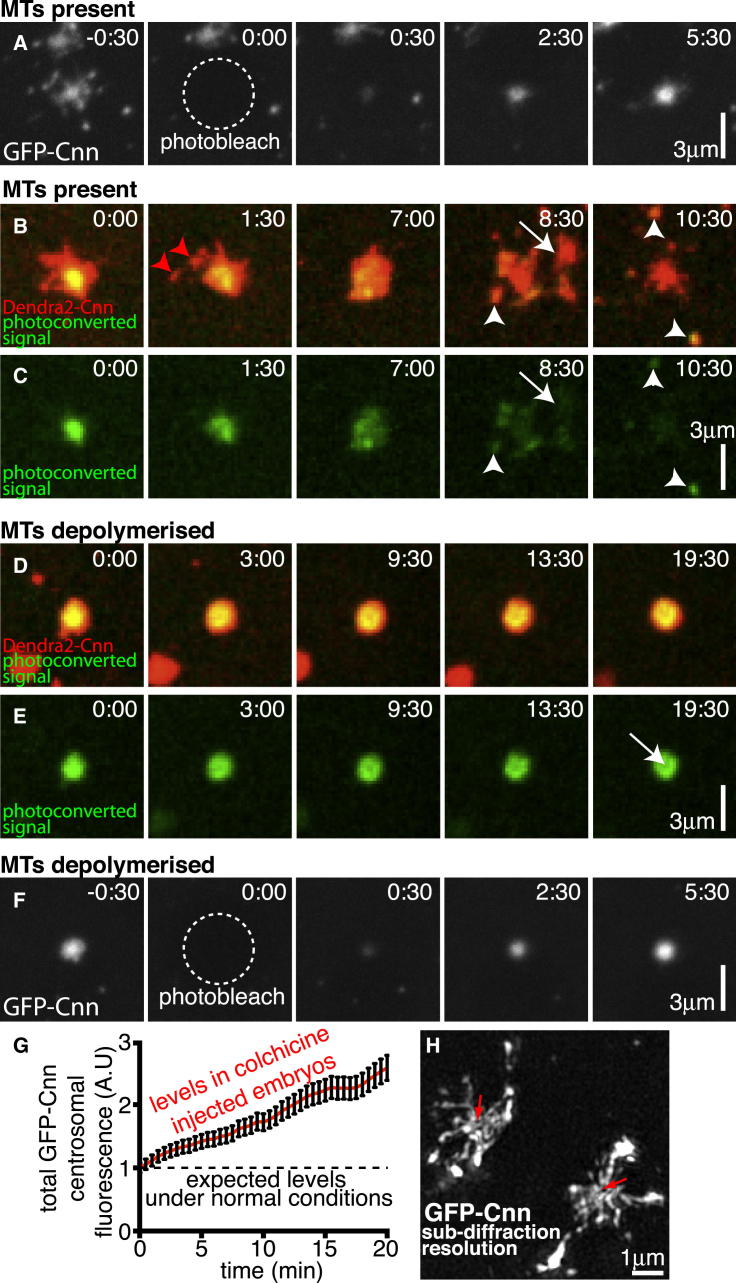
Cnn Initially Incorporates into the Center of the PCM and Then Spreads Outward (A) Confocal images show the behavior of GFP-Cnn before and after photobleaching (t = 0 s) at centrosomes in embryos with intact MTs. (B–E) Confocal images show the behavior of Dendra2-Cnn (pseudo-colored red; B and D) and photoconverted Dendra2-Cnn (pseudo-colored green; B–E) at centrosomes in embryos. Dendra2-Cnn was converted at the center of the PCM at t = 0 and its distribution followed over time in the presence (B and C) or absence (D and E) of MTs. Red arrowheads (B and C; t = 1:30) indicate flares of Dendra2-Cnn at the periphery of the PCM that do not contain any photoconverted protein; white arrowheads (B and C; t = 8:30 and 10:30) indicate flares of Dendra2-Cnn at the periphery of the PCM that now contain photoconverted molecules that were originally generated in the middle of the PCM. The centrosome in (B) and (C) duplicated during the movie and the second centrosome is indicated with an arrow as it moves away (t = 8:30). Note how MT depolymerization (D and E) largely blocks the outward movement of photoconverted Dendra2-Cnn, although a dark hollow develops in the center of the PCM (arrow, E; t = 19:30), indicating that some outward movement has occurred. (F) Confocal images from a FRAP experiment reveal that MT depolymerization does not block the incorporation of GFP-Cnn into the center of the PCM (compare F to A). (G) Graph displays the total levels of unbleached centrosomal GFP-Cnn during M-phase in *Drosophila* embryos where MTs have been depolymerized (red line). The dotted black line indicates the maximal levels of centrosomal GFP-Cnn during a normal mitosis (that normally only lasts 3–4 min and during which time GFP-Cnn levels remain constant; Conduit et al., 2010); MT depolymerization leads to M-phase arrest, allowing measurements to be taken for a longer period, and Cnn continues to steadily accumulate at centrosomes over this time. (H) A super-resolution 3D-SIM image of centrosomes in a living *cnn* null mutant embryo injected with mRNA encoding GFP-Cnn. Red arrows indicate hollows that likely contain a centriole. Error bars = SE. See also Movies S1 and S2.

We used fluorescence recovery after photobleaching (FRAP) to examine the spatiotemporal dynamics of GFP-Cnn incorporation into mitotic centrosomes in *Drosophila* syncytial embryos. As we had observed previously (Conduit et al., 2010), prior to photobleaching, GFP-Cnn was broadly distributed throughout the PCM (Figure 1A; Movie S1A available online, t = −30 s), whereas after photobleaching, GFP-Cnn fluorescence recovered first in the center of the PCM and then gradually spread outward over time (Figure 1A; Movie S1A; t = 30–210 s). Although these observations suggest that new GFP-Cnn molecules bind only around the centrioles and then gradually spread outward in the PCM, it is possible that they bind throughout the PCM but that the rate of exchange in the center of the PCM is faster than that in the periphery, which could give the illusion of outward spread through the PCM.

To distinguish between these possibilities, we expressed Cnn tagged with the photo-switchable protein Dendra2 (Dendra2-Cnn, pseudocolored red) and photoconverted the fluorescence signal (pseudocolored green) specifically in the center of the PCM (Figures 1B and 1C; Movie S2A). If the pattern of GFP-Cnn fluorescence recovery observed in Figure 1A was simply due to differences in exchange rates between the center and periphery of the PCM, the photoconverted signal in the center of the PCM (Figures 1B and 1C and Movie S2A; t = 0:00) would simply dissipate as the photoconverted PCM molecules return to the cytosol. We found, however, that the photoconverted molecules spread slowly outward through the PCM and were replaced in the center by newly incorporated unconverted molecules from the cytosol (Figures 1B and 1C and Movie S2A; t = 0:00 to t = 7:00). The photoconverted molecules ultimately detached from the periphery of the centrosome as PCM “flares” (arrowheads, Figures 1B and 1C and Movie S2A; t = 8:30 to t = 10:30).

These flares have previously been shown to move along centrosomal MTs (Lee et al., 2001; Megraw et al., 2002), so we wondered if the outward movement of Cnn was dependent on MTs. Depolymerizing MTs with colchicine prior to photoconversion strongly inhibited the outward spread of the photoconverted Cnn molecules, particularly at the periphery of the PCM; as a result, the photoconverted signal remained concentrated in the center of the PCM and was no longer lost from the periphery (Figures 1D and 1E; Movie S2B). Even when MTs were depolymerized, however, a dark “hollow” usually appeared at the center of the photoconverted Dendra2-Cnn signal (arrow, Figure 1E; t = 19:30), suggesting that Cnn molecules can initially spread outward a short distance in the absence of MTs; this also appeared to be the case in FRAP experiment movies (compare Movie S1A and Movie S1B). Depolymerizing MTs, however, had little effect on Cnn incorporation into the center of the PCM (compare Figure 1A to Figure 1F and Movie S1A to Movie S1B), so the levels of Cnn at unbleached centrosomes continued to increase steadily over time in embryos injected with colchicine (Figure 1G). Together, these observations demonstrate unambiguously that Cnn molecules continually flux from the center to the periphery of the PCM.

To examine the architecture of the GFP-Cnn molecules within the PCM in more detail we used live, three-dimensional-structured illumination super-resolution microscopy (3D-SIM), which has approximately twice the resolving power of standard confocal microscopes. This revealed that GFP-Cnn formed an extended, scaffold-like structure that appeared to emanate from the centrioles, which were often apparent as a clear hollow at the center of the structure (red arrows, Figure 1H). For reasons described below, we hereafter refer to this structure as the Cnn scaffold.

### Cnn Appears to Be Phosphorylated Specifically at Centrosomes during Mitosis

**Figure 2:**
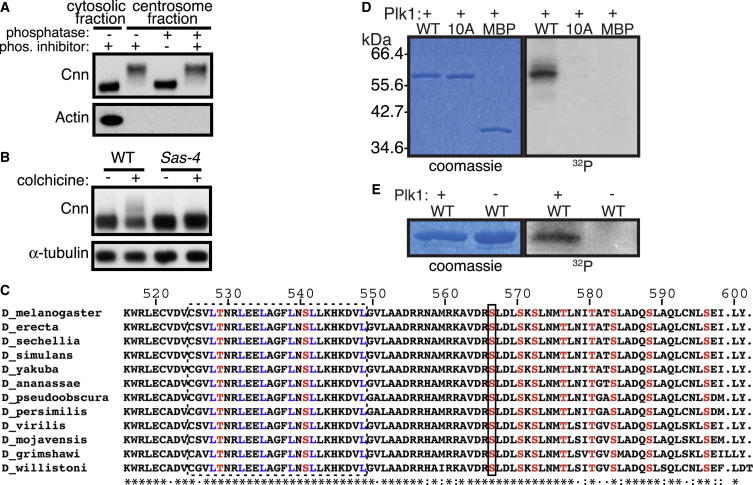
Polo/Plk1 Appears to Phosphorylate Cnn Specifically at Centrosomes (A) Western blot of cytosolic (lane 1) and centrosomal (lanes 2–4) fractions of embryo extracts probed for Cnn (top panel) and Actin (bottom panel). Treatment of the extracts with (+) or without (−) either alkaline phosphatase (phosphatase) or a cocktail of phosphatase inhibitors (phos. inhibitor) is indicated. Cnn displays a mobility shift in the centrosome fraction (lane 2), which is abolished after phosphatase treatment (lane 3), but not if phosphatase inhibitors are included (lane 4). (B) Western blot of interphase (−colchicine) or mitotic (+colchicine) extracts of larval brains from wild-type (WT, lanes 1 and 2) or *Sas-4* mutants (lanes 3 and 4); α-tubulin is shown as a loading control. Some of the Cnn displays a mobility shift in WT mitotic extracts (lane 2) that is not seen in *Sas-4* mutant extracts (lane 4), indicating that the shift is dependent on centrosomes. (C) Alignment of the Cnn PReM domain from *D*. *melanogaster* (K516-Y601) and various other *Drosophila* species. This domain contains a predicted leucine zipper (dotted line box, Leu residues in blue) and ten conserved Ser/Thr residues (in red); the black box indicates S567, identified as a phosphorylation site by MS. Residue numbers for *D*. *melanogaster* are indicated. (D and E) Coomassie-stained gels (left) and autoradiograms (right) from an in vitro kinase assay with (+) or without (−) recombinant human Plk1, containing either WT MBP-Cnn_462-608_ (WT) or mutant MBP-Cnn_462-608_ in which all ten conserved Ser/Thr residues have been mutated to Ala (10A). Only the WT protein is phosphorylated by Plk1; note that phosphorylation leads to only a very small mobility shift in these fragments. See also Table S1.

To understand how fast-moving cytosolic Cnn molecules might be converted into the slow-moving Cnn molecules of the Cnn scaffold, we tested whether Cnn becomes biochemically modified as it incorporates into centrosomes. We compared the electrophoretic mobility of Cnn on western blots of cytosolic and centrosome-enriched fractions from fly embryos. All the Cnn protein in the centrosomal fractions exhibited a mobility shift that could be attributed to protein phosphorylation, and the shift was not detectable in the cytosolic fractions, suggesting that phosphorylation only occurred at centrosomes (Figure 2A). In support of this conclusion, some Cnn protein also showed a mobility shift in mitotic larval brain extracts generated by treating wild-type brains with colchicine (to arrest cells in mitosis and therefore increase the proportion of Cnn molecules at centrosomes); this shift was not detectable in mitotic extracts generated from *Sas-4* mutant brains (which lack centrosomes; Figure 2B), even though these brains are known to be highly enriched for mitotic cells (Basto et al., 2006). Thus, Cnn appears to be phosphorylated specifically at centrosomes during mitosis, potentially explaining why it assembles into a scaffold only around the centrioles.

### Identification of Plk1 Phosphorylation Sites in a Cnn Domain

To identify potential centrosome-specific phosphorylation sites in Cnn, we immuno-isolated Cnn from either cytosolic or centrosome-enriched embryo fractions and performed a tandem mass spectrometry (MS/MS) analysis after enrichment for phosphopeptides. We identified several phosphopeptides in the centrosomal fractions that were not found in the cytosolic fractions (Table S1). Polo kinase is required for the centrosomal recruitment of Cnn during mitosis in *Drosophila* (Dobbelaere et al., 2008; Fu and Glover, 2012), and one of the centrosome-specific Cnn phosphorylation sites (S567) closely conformed to a Polo/Plk1 recognition motif (Santamaria et al., 2011). Moreover, S567 was located within a stretch of 85 amino acids (Lys^516^ to Tyr^601^) that is highly conserved in *Drosophila* and contains nine additional conserved Ser/Thr residues, five of which at least partially conform to the Plk1 recognition motif (Figure 2C). Recombinant human Plk1 could phosphorylate maltose binding protein (MBP)-fusions that included this region in vitro, but not when these ten conserved Ser/Thr residues were mutated to Ala, demonstrating that Plk1 can directly phosphorylate one or more of these sites (Figures 2D and 2E). For reasons explained below, we hereafter refer to this region as the phosphoregulated-multimerization (PReM) domain.

### Phosphorylation of the PReM Domain Allows Cnn to Assemble into a Scaffold around the Centrioles

**Figure 3:**
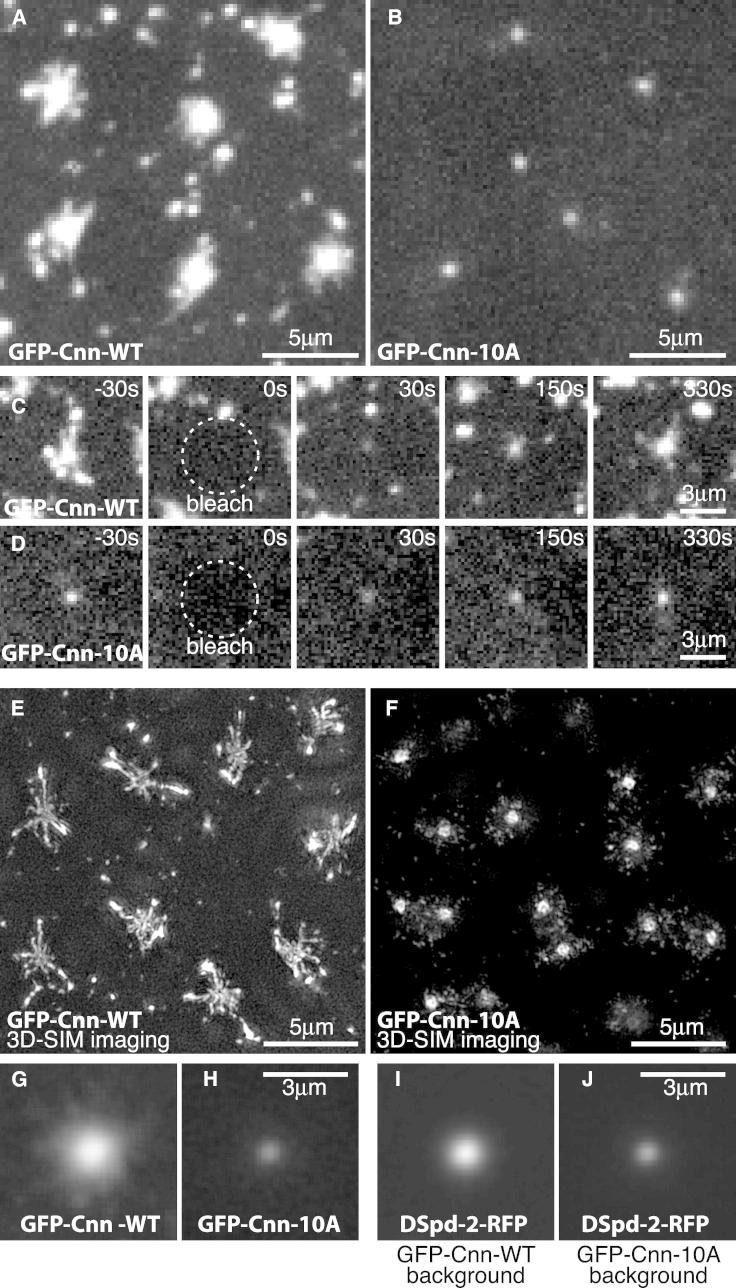
Phosphorylation of the PReM Domain Is Essential for Cnn Scaffold Formation and Efficient PCM Assembly (A and B) Confocal images show centrosomes in *cnn* null mutant embryos injected with mRNA encoding either GFP-Cnn-WT (A) or GFP-Cnn-10A (B). (C and D) Confocal images from a FRAP experiment show the dynamic behavior of GFP-Cnn-WT (C) and GFP-Cnn-10A (D) at centrosomes in embryos lacking endogenous Cnn. Time before and after photobleaching at t = 0 is indicated. (E and F) 3D-SIM images of centrosomes in living *cnn* null mutant embryos injected with mRNA encoding either GFP-Cnn-WT (E) or GFP-Cnn-10A (F). (G–J) Confocal images show the average centrosomal fluorescence of WT GFP-Cnn (G) and GFP-Cnn-10A (H) or DSpd-2-RFP in either a WT GFP-Cnn (I) or a GFP-Cnn-10A (J) background after multiple standard confocal images had been centered and averaged through the Z-plane. Note that the centrosomal level of DSpd-2-RFP and its extent of spread through the PCM are reduced in the GFP-Cnn-10A background. See also Movie S3.

To test whether phosphorylation of the Cnn PReM domain is required for the formation of a centrosomal Cnn scaffold we synthesized mRNAs in vitro encoding GFP fusions with either wild-type (WT) Cnn (GFP-Cnn-WT) or a form of Cnn in which all ten conserved Ser/Thr residues in the PReM domain were mutated to Ala (GFP-Cnn-10A). We injected these mRNAs into early *cnn* null mutant embryos and assayed the behavior of the corresponding proteins 1–2 hr later with confocal imaging. GFP-Cnn-WT exhibited a similar distribution to that of WT GFP-Cnn expressed in WT transgenic embryos (compare Figure 3A to Figure 1A; data not shown), and the GFP-Cnn-WT protein efficiently rescued the *cnn* mutant embryo phenotype. In contrast, although GFP-Cnn-10A localized to some extent to centrosomes and partially rescued the *cnn* null mutant phenotype, its centrosome localization was much weaker than that of GFP-Cnn-WT, and it was concentrated in a much narrower region around the centrioles (compare Figure 3A to Figure 3B and Movie S3A to Movie S3B).

FRAP analysis revealed that GFP-Cnn-10A was continuously recruited to centrosomes but, unlike GFP-Cnn-WT, it no longer spread slowly away from the centrioles (Figures 3C and 3D). A 3D-SIM analysis in live embryos confirmed that GFP-Cnn-10A was localized around centrioles but no longer formed an extended scaffold that spread away from the centrioles (Figures 3E and 3F). Importantly, the failure of GFP-Cnn-10A to form an extended scaffold resulted in impaired PCM assembly, as the amount of another centrosomal protein DSpd-2 (DSpd-2-RFP) recruited to centrosomes, and its ability to spread outward from the centrioles, was strongly reduced in the GFP-Cnn-10A background (Figures 3G–3J). We conclude that phosphorylation of the PReM domain is essential for efficient Cnn scaffold formation and for efficient centrosome maturation.

**Figure 4:**
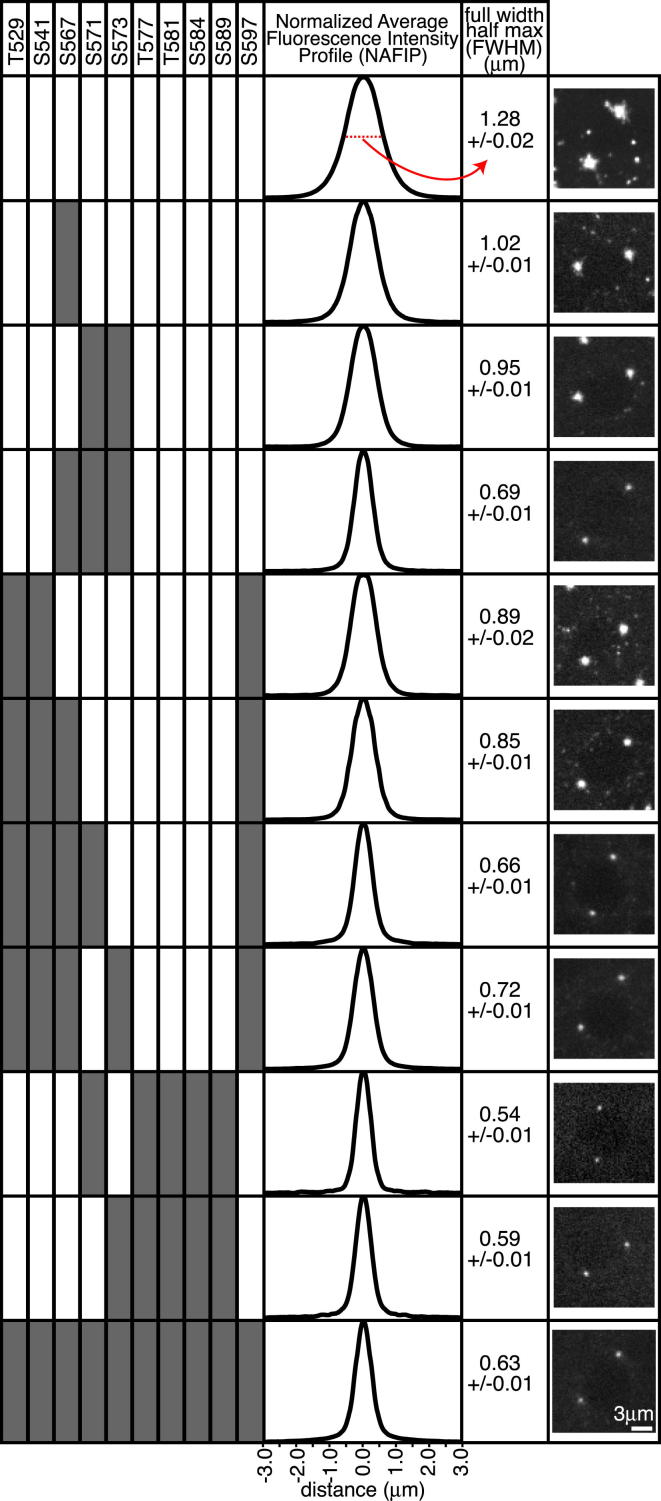
Multiple Phosphorylation Sites within the PReM Domain Regulate Cnn Scaffold Assembly Diagram displays the effect on the centrosomal localization of GFP-Cnn after various Ser/Thr residues have been mutated to Ala (indicated by a gray fill). Graphs show the normalized average fluorescence intensity profile of centrosomes for each combination of mutations (see Supplemental Experimental Procedures); numbers are the average FWHM ± SE of the profiles (giving a quantitative measure of how far each mutant protein spreads out into the PCM); a representative confocal image of each mutant is also shown. In general, the more Ser/Thr residues that are mutated, the less Cnn appears to spread outward from the center of the PCM, although some sites appear to have more influence than others on Cnn scaffold assembly. See also Movie S3.

We used radial profiling to generate normalized average fluorescence intensity profiles (see Supplemental Experimental Procedures) of centrosomes to assess the ability of several mutant forms of Cnn (in which different combinations of the ten Ser/Thr sites were mutated to Ala) to assemble into scaffolds around the centrioles (Figure 4). Many of the Ser/Thr residues appeared to influence scaffold assembly, although some seemed more important than others (for example, mutating S567;S571;S573 gave a stronger phenotype than mutating T529;S541;S597; Figure 4). Moreover, there was a general trend indicating that the more Ser/Thr residues present, the greater the efficiency of scaffold formation (Figure 4). We conclude that PReM domain phosphorylation is not required to recruit Cnn to centrioles, but it is required for Cnn to assemble efficiently into a PCM scaffold that spreads away from the centrioles.

### The PReM Domain Contains a Leucine Zipper that Is Essential for Dimerization In Vitro and Cnn Scaffold Formation In Vivo

**Figure 5:**
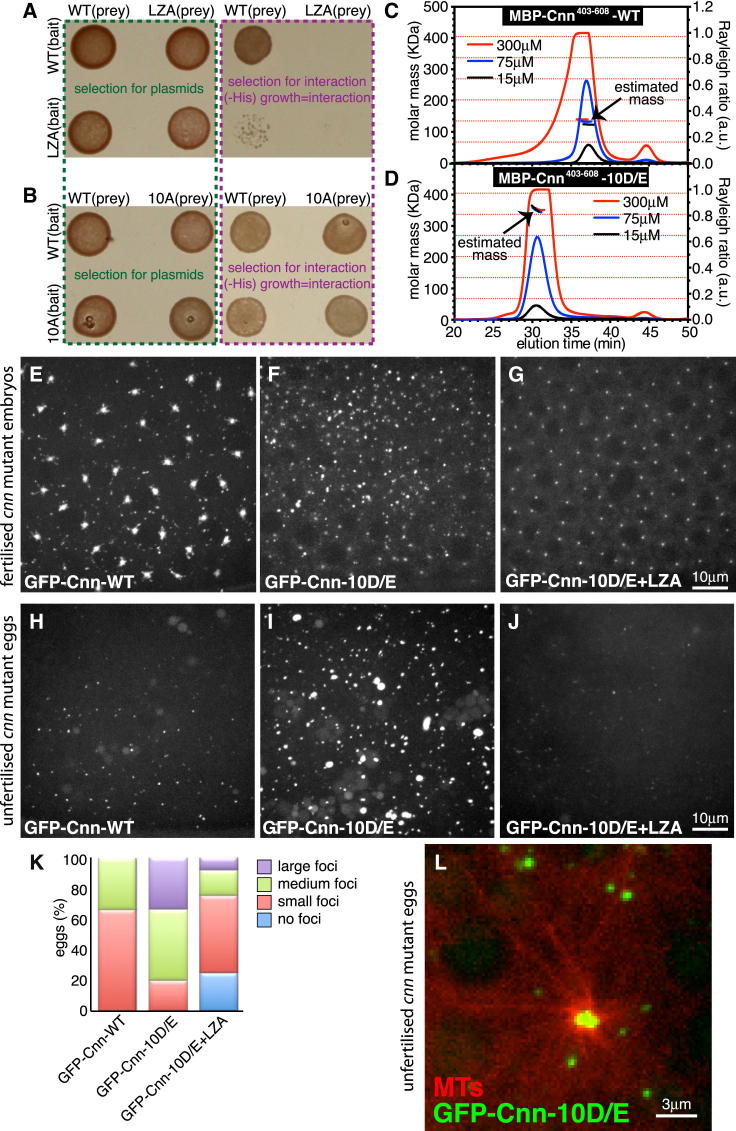
Phosphorylation of the PReM Domain Drives Cnn Scaffold Assembly by Promoting Cnn Multimerization (A and B) A yeast two-hybrid analysis with fragments of Cnn (Cnn_Q403-H608_) containing either a WT PReM domain or a mutated PReM domain, in which either the seven LZ residues (LZA; A) or the ten potentially phosphorylated Ser/Thr residues (10A; B) have been changed to Ala. Left panels show yeast growth on media selecting for the presence of the bait and prey plasmids; right panels show growth on media selecting for an interaction between the bait and prey proteins. The WT and 10A mutants interact with themselves and with each other, whereas the LZA mutant cannot interact with either itself or the WT protein. (C and D) SEC-MALS analysis of MBP-Cnn fragments containing either a WT PReM domain (C) or a mutated PReM domain in which the ten putative phosphorylation sites have been changed to phosphomimicking residues (10D/E; D). An equal volume of protein at either 15 μM (black lines), 75 μM (blue lines), or 300 μM (red lines) concentration was loaded onto the column. Expected masses of a monomer (67.4 KDa, determined by MS) and successive multimers (dimer, trimer, etc.) are indicated with horizontal red dotted lines across the graphs. WT MBP-Cnn has an average mass similar to that of a dimer, whereas MBP-Cnn-10D/E has an average mass most similar to that of a pentamer. Note that the calculated mass of MBP-Cnn-10D/E remains constant across a wide range of protein concentrations, indicating that these multimeric complexes are highly stable. (E–J) Confocal images of *cnn* null mutant embryos (E–G) or unfertilized eggs (H–J) injected with mRNA encoding either GFP-Cnn WT (E and H), GFP-Cnn10D/E (F and I), or GFP-Cnn10D/E+LZA (G and J). Cytosolic foci of WT GFP-Cnn (E) can be seen in the embryos, but these are largely flares that have broken away from the periphery of the PCM (Megraw et al., 2002). GFP-Cnn10D/E forms many more cytosolic foci (F), making it difficult to distinguish the centrosomes; these foci are not formed if the LZ is also mutated, which also blocks Cnn scaffold formation around the centrioles (G). In eggs, which lack centrosomes, GFP-Cnn-WT (H) forms small foci in the cytoplasm, but GFP-Cnn10D/E forms much larger foci (I); these foci are dramatically decreased in size and intensity if the LZ is also mutated (J). (K) Quantification of foci formation in eggs by WT GFP-Cnn, GFP-Cnn10D/E, or GFP-Cnn10D/E+LZA. (L) Confocal image of an unfertilized egg expressing the MT marker Jupiter-mCherry (red) and GFP-Cnn10D/E (green) shows that the larger GFP-Cnn10D/E foci can organize MT asters independently of centrosomes. See also Figure S1 and Movies S3, S4, and S5.

How might the PReM domain allow Cnn to assemble into a scaffold structure? We noticed that the PReM domain contains a leucine zipper (LZ; bold and boxed region in Figure 2C; Heuer et al., 1995). In yeast two hybrid (Y2H) assays, Cnn fragments containing the WT PReM domain strongly self-interacted but not when the seven residues in positions “a” and “d” of the LZ were mutated to Ala (LZA; Figure 5A). Size exclusion chromatography multi-angle light-scattering (SEC-MALS) analyses revealed that purified MBP-Cnn fusions containing the WT PReM domain (MBP-Cnn-WT) existed predominantly as dimers (Figure 5C; Figure S1A), whereas the equivalent MBP-Cnn-LZA fusions existed predominantly as monomers (Figure S1B), strongly suggesting that the LZ is important for PReM domain dimerization in vitro. Interestingly, however, Cnn fragments containing the PReM domain with the Cnn-10A phosphomutations still self-interacted in Y2H assays (Figure 5B) and still behaved predominantly as dimers in vitro (Figure S1C). Thus, the LZ is required for dimerization of the PReM domain in vitro, but this dimerization appears to occur independently of phosphorylation. We found, however, that full-length GFP-tagged Cnn containing the LZ mutations (GFP-Cnn-LZA) had a similar phenotype to the GFP-Cnn-10A mutant—both proteins were recruited to centrioles, but neither protein formed scaffold structures around the centrioles (compare Movie S3B to Movie S3C). Thus, although phosphorylation is not required for LZ-dependent dimerization, the LZ is essential for phosphorylation-driven Cnn scaffold formation in vivo.

### Phosphomimetic Mutations within the PReM Domain Allow Cnn to Multimerize In Vitro and to Form Cnn Scaffolds Spontaneously In Vivo Independently of Centrosomes

To test whether phosphorylation of the PReM domain enables Cnn to form higher-order multimers in vitro, we examined the behavior of MBP-Cnn fusions containing phosphomimetic mutations in the PReM domain (MBP-Cnn-10E/D). Remarkably, these fusions formed higher-order multimers that had an average mass most consistent with that of a pentamer (Figure 5D; Figure S1D). The average mass of these multimers did not change over a wide range of protein concentrations, suggesting that they had a relatively stable structure (Figure 5D). The phosphomimetic multimers reverted to a predominantly monomeric state if the LZ was also mutated (MBP-Cnn-10E/D-LZA), demonstrating that multimer formation is also dependent on the LZ (Figure S1E). Thus, phosphomimetic mutations within the PReM domain allow MBP-Cnn fusions to form LZ-dependent higher-order multimers in vitro.

We tested whether phosphomimetic mutants of full-length Cnn (GFP-Cnn-10E/D) would form Cnn scaffolds around the centrioles in vivo more efficiently than GFP-Cnn-WT. Using the assay described earlier, in which mRNA is injected into *cnn* mutant embryos, we found the opposite: GFP-Cnn-10E/D was recruited to centrosomes but less efficiently than GFP-Cnn-WT (compare Figure 5E to Figure 5F). However, this appeared to be because GFP-Cnn-10E/D also spontaneously formed many prominent foci in the cytoplasm, independently of centrosomes (Figure 5F), and these foci competed with centrosomes for the GFP-Cnn-10E/D protein, as they gradually increased in brightness over time (their presence often eventually making it difficult to detect the real centrosomes; Figure 5F; Movie S4A). The formation of these cytosolic foci was abolished if the LZ was also mutated (GFP-Cnn-10E/D-LZA; Figure 5G), and this protein localized to the centrosomes in a similar manner to GFP-Cnn-10A and GFP-Cnn-LZA (Figure 5G; Movies S3B and S3C), strongly suggesting that these cytoplasmic foci use the same LZ-dependent assembly pathway as the centrosomal Cnn scaffolds.

To confirm that GFP-Cnn-10E/D could form scaffold structures independently of centrosomes, we injected its mRNA into unfertilized eggs, which lack centrosomes. Whereas GFP-Cnn-WT usually formed small foci in eggs, GFP-Cnn-10E/D usually formed much larger foci (compare Figures 5H, 5I, and 5K; Movie S4B), and foci formation was strongly reduced if the LZ was also mutated (Figures 5J and 5K). Most strikingly, the larger GFP-Cnn-10E/D foci often organized dynamic MT asters in the unfertilized eggs (Figure 5L; Movie S5), indicating that the phosphomimetic Cnn scaffold can organize MTs even when it assembles independently of centrosomes. We conclude that phosphomimetic mutations of the PReM domain dramatically increase the efficiency of Cnn scaffold formation in vivo. This presumably explains why GFP-Cnn-10E/D can assemble into scaffolds independently of centrosomes, because it no longer requires phosphorylation at the centrosome to convert it into an assembly-competent form.

## Discussion

**Figure 6:**
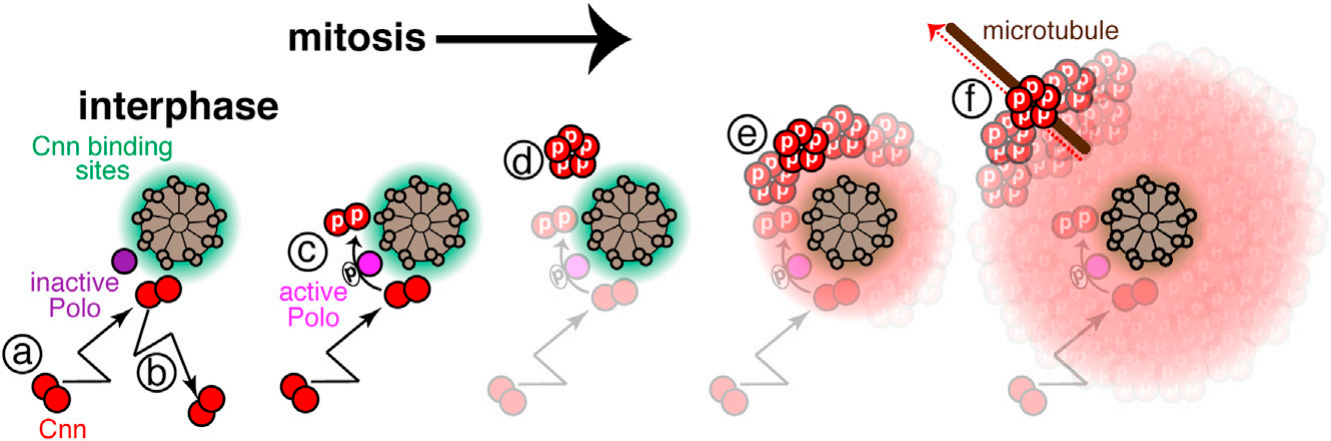
A Schematic Model of Cnn Scaffold Assembly In the cytosol, Cnn molecules (red circles) exist predominantly as dimers (a), which form via the LZ of the PReM domain. Centriole binding sites for Cnn (*green haze*) may be present in interphase centrioles, as some fly cells, such as cultured S2 cells, can organize small amounts of Cnn and PCM during interphase (Mennella et al., 2012). Because centriole-associated Polo is inactive, however, Cnn is not phosphorylated and therefore cannot assemble into a scaffold structure around the interphase centrioles; the Cnn molecules released from their centriole binding sites therefore immediately return to the cytosolic fraction (b). As cells enter mitosis, centriole-associated Polo is activated and phosphorylates the Cnn PReM domain (c), promoting multimerization through the LZ (d). The Cnn multimers (here depicted as pentamers, based on our in vitro SEC-MALS data) can further interact with one another through different regions of Cnn and thereby assemble into a macromolecular scaffold (e), which can only move slowly away from the centrioles. The initial short-range movement of the scaffold away from the centrioles appears to be MT-independent, but the outward movement at the periphery of the PCM is strongly dependent on MTs (f). Thus, the mitotic Cnn scaffold is in flux, as it continuously assembles around the centrioles and disassembles at the periphery of the PCM, most likely because the Cnn molecules eventually become dephosphorylated at the periphery and so lose their ability to multimerize. In this way, Cnn assembles from the inside out to form a scaffold around the centrioles. This expanded scaffold helps recruit other PCM proteins, thus explaining why centrosomes increase in size so dramatically (mature) during mitosis. See also Table S2.

As cells enter mitosis, centrosomes mature, and the amount of PCM recruited around the centrioles dramatically increases (Palazzo et al., 2000). Although many proteins have been implicated in this process, we know little about how they organize a functional mitotic centrosome. Previous studies have hinted at the existence of a PCM scaffold, but its molecular nature has remained elusive (Dictenberg et al., 1998; Schnackenberg et al., 1998). Our data suggest that Cnn is phosphorylated specifically at centrosomes during mitosis, and this phosphorylation allows Cnn to assemble into a scaffold around the centrioles (Figure 6). Perturbing Cnn phosphorylation prevents efficient scaffold assembly and efficient mitotic PCM recruitment, demonstrating that the phosphorylated Cnn scaffold plays an important part in centrosome maturation in flies.

We demonstrate unambiguously that the Cnn scaffold is in constant flux: as the Cnn scaffold spreads slowly outward, it is continuously replenished by new phosphorylated Cnn that assembles around the centrioles; in this way, the Cnn scaffold is built from the inside out. This inside-out assembly mechanism has important implications, because it potentially explains how centrioles can influence the size of the PCM (Conduit et al., 2010) and organize centrosomes of different sizes within the same cell (Conduit and Raff, 2010)—as seems to occur in several asymmetrically dividing stem/progenitor cells (Lesage et al., 2010; Nigg and Stearns, 2011; Pelletier and Yamashita, 2012).

How does Cnn assemble into a scaffold structure? We show that Cnn contains a PReM domain that contains a LZ and ten Ser/Thr residues that are highly conserved in *Drosophila* species. Mutating the LZ or the ten Ser/Thr residues to Ala strongly inhibits Cnn scaffold assembly in vivo, while mutating these ten Ser/Thr residues to phosphomimicking residues promotes spontaneous Cnn scaffold assembly in the cytosol, independently of centrosomes. Moreover, whereas the WT PReM domain predominantly forms dimers via the LZ in vitro, replacing the ten Ser/Thr residues with phosphomimicking residues allows the PReM domain to assemble into higher-order multimers in an LZ-dependent manner. Our modeling suggests that the arrangement of hydrophobic and hydrophilic residues within the LZ could allow multiple LZs to associate laterally to form such multimeric structures (unpublished data). We speculate, therefore, that these stable multimers formed by the phosphomimicking mutant PReM domains in vitro may be the fundamental building blocks of the phosphorylated Cnn scaffold in vivo (Figure 6). How these multimers assemble into a larger macromolecular scaffold is unclear, but our Y2H analysis indicates that multiple regions of Cnn can self-interact and so could potentially participate in such a process (Table S2; Figure 6).

How is Cnn scaffold assembly regulated so that it only occurs during mitosis? Polo/Plk1 is a key regulator of PCM assembly in many systems (Barr et al., 2004; Blagden and Glover, 2003; Haren et al., 2009) and it is activated in human cells during the G2/M transition (Petronczki et al., 2008; Seki et al., 2008). In flies, knocking down Polo in cultured fly cells abolishes Cnn phosphorylation (Dobbelaere et al., 2008) and strongly perturbs Cnn’s centrosomal localization (Dobbelaere et al., 2008; Fu and Glover, 2012). We show here that recombinant human Plk1 can phosphorylate the PReM domain of Cnn in vitro (Figures 2D and 2E) and that at least six of the putative phosphorylation sites within the PReM domain conform to a Polo/Plk1 recognition motif. Moreover, abolishing these putative phosphorylation sites prevents Cnn phosphorylation in vitro and Cnn scaffold formation in vivo, whereas mutating these sites to phosphomimicking residues promotes multimerization in vitro and spontaneous scaffold formation in vivo. Thus, it seems likely that Polo is activated during mitosis in fly cells and directly phosphorylates Cnn to initiate Cnn scaffold assembly (Figures 6A and 6B), although we cannot exclude the possibility that Polo activates an unknown kinase that then phosphorylates Cnn.

How is Cnn scaffold assembly regulated so that it only occurs around the centrioles? Our data strongly indicate that Cnn is normally phosphorylated exclusively at centrosomes, and Polo is highly concentrated at centrioles throughout the cell cycle (Fu and Glover, 2012). While it remains formally possible that Cnn is phosphorylated in the cytosol and phosphorylated Cnn is then rapidly sequestered at centrosomes, we think this unlikely for two reasons: (1) phosphomimetic Cnn is not rapidly transported to centrosomes, but rather spontaneously assembles into scaffolds in the cytoplasm, and (2) in mitotic extracts of brain cells that lack centrosomes, we cannot detect any phosphorylated Cnn (Figure 2B). It is interesting that the phosphorylation of at least six of the ten conserved Ser/Thr residues within the PReM domain appears to be required for efficient scaffold assembly (Figure 4). The potential advantages of regulation by multisite phosphorylation in allowing switch-like transitions are well documented (Salazar and Höfer, 2009). Thus, it seems likely that the requirement for multisite phosphorylation helps ensure that Cnn normally only efficiently forms a scaffold around the centrioles, where there is a high concentration of both the kinase and its substrate.

Cnn is a large protein that contains several predicted coiled-coil regions, supporting the idea that it can act as a molecular scaffold onto which other PCM proteins can assemble. Proteins related to Cnn have been identified in species ranging from yeasts to humans, and many of these proteins have been implicated in centrosome or MT organizing center assembly (Barr et al., 2010; Choi et al., 2010; Lizarraga et al., 2010; Samejima et al., 2010); they are also usually large proteins with several predicted coiled-coil domains, and some family members have been shown to interact directly with several other PCM components, including the γTuRC (Choi et al., 2010; Samejima et al., 2008; Terada et al., 2003), Aurora A (Terada et al., 2003), and Pericentrin (Buchman et al., 2010; Wang et al., 2010). Although we have been unable to identify an obvious PReM domain in vertebrate Cnn family members, many of these proteins have regions that might fulfill the minimal requirements for a PReM-like domain—a potential coiled-coil interaction domain, and a region containing multiple potential phosphorylation sites. We therefore suspect that Cnn-like proteins will contribute to PCM scaffold formation in many systems.

## Experimental Procedures

### Transgenic *Drosophila* Lines

The Ubq-Cnn-Dendra2 and Ubq-RFP-DSpd-2 P-element-mediated transformation vectors were made by introducing full-length Cnn cDNA or DSpd-2 cDNA into the Ubq-Dendra2NT or Ubq-RFPNT Gateway vector, respectively (Basto et al., 2008). Transgenic lines were generated by Bestgene (USA). GFP-Cnn (Lucas and Raff, 2007) and Jupiter-mCherry (Callan et al., 2010) have been described previously.

### Dynamic Analysis of GFP and Dendra2 Fusion Proteins

Syncytial stage embryos were imaged on a Perkin Elmer ERS Spinning Disk confocal system (ERS software) mounted on a Zeiss Axiovert microscope, using a 63×, 1.4NA oil-immersion objective. FRAP analysis was carried out during S phase of cycle 11 or 12. We collected 0.5 μm thick confocal sections through the center of a selected centrosome. We bleached GFP signals using a focused 440 nm laser. We converted the Dendra2 signal using a focused 405 nm laser (targeted specifically at the central 4 pixels of the centrosome).

### 3D-Structured Illumination Microscopy

Embryos from *cnn*^*f04547*^/*cnn*^*HK21*^ hemizygous mutant mothers were injected with mRNA encoding either WT GFP-Cnn or GFP-Cnn10A and imaged at 21°C on an OMX V3 microscope (Applied Precision) with a 60×/1.35 NA oil-immersion objective (Olympus). Images were processed using SoftWorx software (Applied Precision). Images shown are maximum intensity projections of several z-slices.

### Production of Centrosome and Cytosolic Fractions and Phosphatase Treatment

Whole centrosomes were isolated from embryonic extracts using a modified version of a centrosome isolation protocol (Lehmann et al., 2006). Briefly, embryo extract containing 50% sucrose was layered on top of a sucrose cushion consisting of 55% and 70% sucrose. The tubes were spun at 27,000 rpm, causing the centrosomes in the extract to move into the 70% sucrose layer. “Cytosolic” and “centrosome” fractions were collected from the top and bottom of the tube, respectively. Phosphatase treatment was carried out using alkaline phosphatase (Roche) for 4.5 hr at 37°C with or without phosphatase inhibitor cocktails 2 and 3 (Sigma).

### Analysis of Mitotic and Nonmitotic Brain Extracts

WT or *Sas-4* mutant third instar larval brains were dissected and incubated in Schneider’s Insect Medium (Sigma) supplemented with 10% fetal bovine serum (Sigma) and Pen/Strep (Sigma), either with or without 1.25 mM colchicine for 6 hr at 25°C. The brains were then boiled in 20 μl 1× sample buffer containing phosphatase inhibitor cocktails 2 and 3 (Sigma) and the extracts were run on a 3%–8% polyacrylamide gel.

### Centrosome Immunoprecipitation and Mass Spectrometry

Briefly, centrosomal or cytosolic Cnn molecules were immunoprecipitated using rabbit anti-Cnn antibodies coupled to protein A conjugated magnetic Dynabeads (Life Technologies). Centrosomal and cytoplasmic fractions (obtained as described above) were diluted and rotated with the antibody beads at 4°C for 2 hr. Beads were washed, boiled in SB, and separated from the sample using a magnet. The samples were separated on a polyacrylamide gel and the band containing Cnn was cut out and treated as described in the Supplemental Experimental Procedures. Gel pieces were incubated overnight with Promega sequencing grade modified trypsin. Formic acid was added to stop the digestion. Supernatant containing the peptides was transferred to a new tube and the gel pieces were incubated with extraction buffer to extract any remaining peptides. Samples were dehydrated using a vacuum concentrator and enriched for phosphopeptides as described in the Supplemental Experimental Procedures. Liquid chromatography-MS/MS analysis was performed using a LTQ Orbitrap Mass Spectrometer (Thermo Scientific) coupled to a UltiMate 3000 Nano LC system (Thermo Scientific).

### Image Analysis

We used Image J to calculate the average centrosomal fluorescence profile for the different Cnn phosphorylation mutants. At least 16 centrosomes (28 centrosomes on average) from several embryos were used to calculate the average distribution for each protein type. To calculate the profile for an individual centrosome, we first calculated the center of mass of the centrosome by thresholding the image and running the “analyze particles” (center of mass) macro on the most central Z plane of the centrosome. We then centered concentric rings (spaced at 0.028 μm and spanning across 3.02 μm) on this center and measured the average fluorescence around each ring (radial profiling). After subtracting the average cytosolic signal, an average profile for the given protein type was calculated. This profile was normalized so the peak intensity was equal to 1, and the profile was mirrored to show a full symmetric centrosomal profile.

To calculate the FULL WIDTH HALF MAX (FWHM) for each form of GFP-Cnn, a normalized mirrored profile was calculated for each individual centrosome. Each profile was analyzed with the ImageJ Gaussian curve-fitting macro to produce a “d” value. This “d” value was multiplied by 2.35482005 to produce a FWHM value for the profile. The average FWHM value and its SE for each form of GFP-Cnn was then calculated to produce an overall FWHM ± SE value.

For producing average fluorescence images of GFP-Cnn-WT and RFP-DSpd-2 (from 29 centrosomes) and GFP-Cnn-10A and RFP-DSpd-2 (from 25 centrosomes), we first aligned the centrosomes by producing a stack of images where the center of mass of each centrosome was positioned in the center of the cropped image. We then generated an average Z-projected image.

### Yeast Two-Hybrid Analysis

All yeast two-hybrid experiments were carried out using pPC86-AN and pPC97-AN vectors; the Y8800 and Y8930 yeast strains were kindly supplied by Mike Boxem (Boxem et al., 2008). For assays testing the effect of mutating the LZ or the effect of mutating phosphorylation sites with the PReM domain on the self-interaction of Cnn fragments, both baits and prey fragments encoded the region of Cnn from Q403 to H608. For assays testing Cnn-Cnn interactions in general, the bait fragments encoded the N-terminal, middle, and C-terminal thirds of Cnn, or the N-terminal two-thirds, C-terminal two-thirds of Cnn, and the full-length Cnn protein; the preys encoded smaller ∼200 amino acid fragments and larger combinations of these fragments (Table S2).

### GFP-Cnn Foci Quantification

Images from eggs expressing WT GFP-Cnn (15 eggs), GFP-Cnn10D/E (15 eggs), or GFP-Cnn10D/E+LZA (12 eggs) were scored blind for quantification. The images from each genotype were compiled, numbered randomly, and then scored by three individuals (who were not involved in obtaining or numbering the images) as containing no foci, small foci, medium foci, or large foci. The consensus phenotype (the phenotype scored by at least two people) was taken as the true phenotype for each egg.

### Statistical Analysis

Error bars above and below the mean value in Figure 1G and FWHM error values in Figure 4 represent the SEM as calculated by dividing the SD by the square root of n.

More extensive details of our experimental procedures, including details of antibodies used, can be found in the Supplemental Experimental Procedures.

## Author Contributions

P.T.C. and J.W.R. devised the experiments and wrote the manuscript; Z.F. performed the kinase assays and MALS analysis with S.J. and S.M.L.; J.H.R. performed the Y2H analysis; J.B. carried out mass spectrometry with P.T.C.; A.W. performed the 3D-SIM analysis; S.D.B. helped analyze the protein dynamics data; J.D. initiated the studies on Cnn phosphorylation; P.T.C. carried out all other experiments.

## Supplemental Information

Document S1. Supplemental Experimental Procedures and Figure S1

Document S2. Tables S1 and S2

Movie S1, Related to Figure 1. GFP-Cnn Molecules Are Initially Incorporated into the Center of the PCM in an MT-Independent Manner These movies show the results of FRAP experiments illustrating the dynamic behavior of GFP-Cnn at centrosomes in *Drosophila* embryos with either intact MTs (A) or MTs depolymerized by colchicine injection (B). Time before and after photobleaching at t = 0 is shown at the top right of each panel. Note how, in (A), the GFP-Cnn fluorescence signal initially recovers in the center of the PCM and then spreads outward; in (B), in the absence of MTs, the rate of GFP-Cnn incorporation into the center of the PCM is largely unperturbed, but the outward spread into the periphery is decreased.

Movie S2, Related to Figure 1. GFP-Cnn Molecules Spread Outward through the PCM in a Largely MT-Dependent Manner These movies show the results of photoconversion experiments illustrating the dynamic behavior of Dendra2-Cnn (pseudocolored red) at centrosomes in *Drosophila* embryos with either intact MTs (A) or MTs depolymerized by colchicine injection (B). Time from when the molecules in the central region of the centrosome had been photoconverted (pseudocolored green) is shown at the top right of each panel. In each case, photoconversion was carried out in S phase, and the centrosomes were filmed through M phase. When MTs are intact (A), the photoconverted signal gradually spreads outward through the PCM during S phase (t = 0 to t = 2.5). During M phase (t = 3 to t = 6), the movement slows down—Cnn flaring is known to be suppressed during mitosis (ref)—but, when the embryo enters the following S phase (t = 6.5), flaring resumes and the photoconverted molecules rapidly spread outward and are lost from the PCM as flares. This interpretation is consistent with the behavior of photoconverted molecules after MTs have been depolymerized (B), in which the photoconverted molecules initially move outward a short distance (explaining the presence of the dark hollow that becomes weakly visible at the center of the photoconverted molecules) but then remain clustered in the center of the PCM.

Movie S3, Related to Figure 3. Cnn Scaffold Formation Is Dependent on Phosphorylation of the PReM Domain and the Presence of an Intact LZ These movies show the localization of GFP-CnnWT (A), GFP-Cnn-10A (B), and GFP-Cnn-LZA (C) at centrosomes in *cnn* null mutant embryos injected with mRNA encoding the respective protein. Time after the start of each movie is shown at the top right of (C). (A) GFP-Cnn WT exhibits a similar localization to that seen with WT GFP-Cnn provided transgenically (see Figure 1A; Movie 1A; data not shown), and the protein appears to efficiently rescue the *cnn* null mutant embryo phenotype (the nuclei and spindles within embryos laid by *cnn* mutant mothers are normally severely misorganized). (B and C) When either the PReM domain phosphorylation sites (B) or the a and d positions within the PReM domain LZ (C) are mutated to alanine, however, the amount of GFP-Cnn at centrosomes is reduced and GFP-Cnn displays a much tighter distribution around the centrioles. The *cnn* null mutant phenotype is at least partially rescued, because the nuclei and spindles are better organized than those of *cnn* null mutant embryos.

Movie S4, Related to Figure 4. GFP-Cnn Foci Form Spontaneously in the Cytosol When Phosphorylation within the PReM Domain Is Mimicked by Mutation These movies show the behavior of GFP-Cnn-10D/E in *cnn* null mutant *Drosophila* embryos (A) or unfertilized eggs (B) injected with mRNA encoding GFP-Cnn-10D/E. Time since the start of each movie is shown at the top right of each panel. (A) In embryos, GFP-Cnn-10D/E localizes to centrosomes but also forms independent cytosolic foci that increase in size over time. The centrosomes can be seen clearly at the beginning of the movie but become increasingly difficult to distinguish as the movie progresses because of buildup of the cytosolic GFP-Cnn-10D/E foci. (B) GFP-Cnn-10D/E foci also form in unfertilized eggs, which lack centrosomes, showing that the foci form spontaneously in the cytosol independently of centrosomes. These foci also increase in size over time. The larger (but more weakly fluorescent) round objects in the unfertilized egg (B) are yolk granules that autofluoresce.

Movie S5, Related to Figure 4. GFP-Cnn10E/D Foci that Form Spontaneously in the Cytosol Can Organize Dynamic MTs This movie shows GFP-Cnn10D/E foci (green) in unfertilized eggs expressing the MT marker Jupiter-mCherry (red). Time since the start of the movie is shown at the top right. At this resolution, individual MTs are impossible to discern, but the MTs organized by the GFP-Cnn10D/E foci appear to be dynamic.
