# Supplementary material for: The Centrosome-Specific Phosphorylation of Cnn by Polo/Plk1 Drives Cnn Scaffold Assembly and Centrosome Maturation
Source: Dev Cell. 2014 Mar 31;28(6):659–69. doi: 10.1016/j.devcel.2014.02.013 (PMC3988887; doi:10.1016/j.devcel.2014.02.013)
Supplement: Document S1. Supplemental Experimental Procedures and Figure S1 [file mmc1.pdf]

Developmental Cell, Volume *28*

Supplemental Information

The Centrosome-Specific Phosphorylation of Cnn by Polo/Plk1 Drives Cnn Scaffold Assembly and Centrosome Maturation

Paul T. Conduit, Zhe Feng, Jennifer H. Richens, Janina Baumbach, Alan Wainman, Suruchi D. Bakshi, Jeroen Dobbelaere, Steven Johnson, Susan M. Lea, and Jordan W. Raff

Supplementary Information

Supplementary Figures

Figure S1

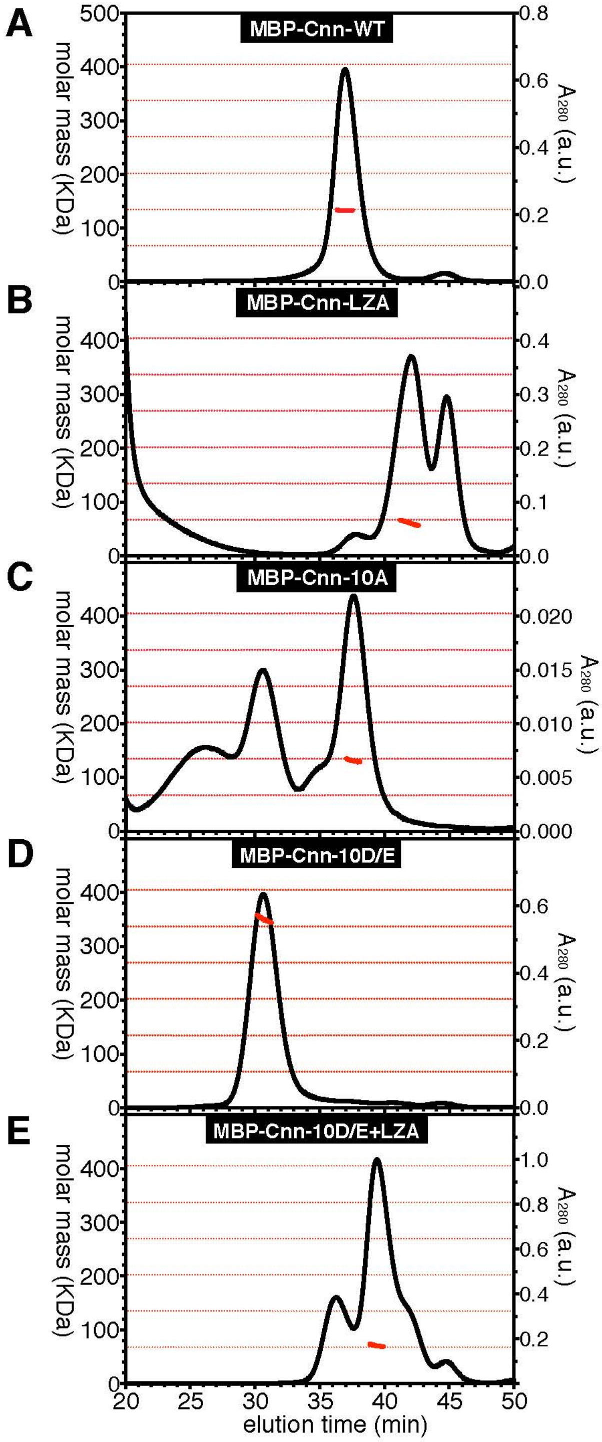

Figure S1, related to Figure 4

SEC-MALS analysis of WT and PReM domain mutants of MBP-Cnn fragments. Graphs show the results of a Size Exclusion Chromatography-Multi Angle Light Scattering (SEC-MALS) analysis of MBP-tagged Cnn fusion proteins. These fusions contain either the WT $Cnn_{Q403\text{-}H608}$ sequence (MBP-Cnn-WT) (**A**), or the same sequence with the following mutations: the 7 *a* and *d* positions of the LZ mutated to alanine (MBP-Cnn-LZA) (**B**); the 10 phosphorylation sites in the PReM domain mutated to Ala (MBP-Cnn-10A) (**C**); the 10 phosphorylation sites in the PReM domain mutated to phospho-mimicking residues (MBP-Cnn-10D/E) (**D**); the 10 phosphorylation sites in the PReM domain mutated to phospho-mimicking residues (MBP-Cnn-10D/E) and the 7 *a* and *d* positions of the LZ mutated to alanine (MBP-Cnn-10D/E+LZA) (**E**). The graphs display the UV absorbance (A_{280}, right hand y-axis) profile of each protein as they elute from a Superose-6 gel filtration column over time (larger complexes pass through the column more quickly, shifting the elution profile to the left). The average mass of the protein complexes in each major peak has been determined by MALS (indicated by the solid red lines underneath each peak) (KDa, left hand y-axis). Dotted red lines across the graph indicate the predicted mass of a monomer (~67.4KD, which was directly confirmed by MS analysis—data not shown) and the predicted mass of successive multimers (dimer, trimer, etc).

MBP-Cnn-WT has an approximate average mass of a dimer (A). The major MBP-Cnn-LZA peak has an approximate average mass of a monomer (B),

indicating that the LZ is required for dimerization. The major MBP-Cnn-10A peak has an approximate average mass of a dimer (C), indicating that phosphorylation is not required for dimerization. The major MBP-Cnn-10D/E peak has an approximate average mass of a pentamer (D), indicating that phospho-mimicking residues can drive multimerisation of the PReM domain. The major MBP-Cnn-10D/E+LZA peak has an approximate average mass of a monomer (E), indicating that the LZ is required for multimerisation. The smaller peak in (B) is likely a degradation product (confirmed by MS; data not shown). Note that large amounts of MBP-Cnn-LZA are found in the void volume—most likely because the exposed hydrophobic residues in the monomer can lead to random aggregation. Note also that there are several overlapping higher mass peaks in (C) that are also likely aggregates of MBP-Cnn-10A caused by the replacement of polar serines and threonines with hydrophobic alanines; this is also indicated by the presence of some MBP-Cnn10A in the void volume.

Supplementary Tables

Supplementary tables can be downloaded as excel files at the bottom of this document.

Table S1, related to Figure 2

Mass spectrometry analysis of centrosomal and cytosolic extracts. This table shows the Mascot results of a mass spectrometry analysis performed on centrosome and cytosolic fractions from *Drosophila* embryos. Only top ranked peptides with an expected score of <0.05 are reported. Note how 9 sites (highlighted by blue boxes) were found only in the centrosomal fraction.

Table S2, related to Figure 7

A yeast-2-hybrid analysis testing the interactions between various Cnn fragments. A yeast-2-hybrid analysis was carried out using various bait and prey fragments of Cnn (as indicated in columns A and B; a list of all bait and prey fragments tested is shown in columns I and J; fragments containing the PReM domain are highlighted in red). Three different reporters were tested – His (column C), Ade (column E), and LacZ (column G). Interaction levels are indicated as strong, medium, weak or none. All combinations of baits and preys were tested, but only those that scored positive in at least one assay are shown.

Supplementary Experimental Procedures

Fly Stocks

For analysing GFP-Cnn centrosomal dynamics, we analysed embryos from mothers expressing 1 copy of Ubq-GFP-Cnn in a *cnn*^{f04547}/*cnn*^{HK21} hemizygous mutant background (Lucas and Raff, 2007). For analyzing Dendra2-GFP centrosomal dynamics we analysed embryos from mothers expressing 2 copies of Dendra2-Cnn in a WT background. For examining the effect of colchicine injection on GFP-Cnn centrosomal dynamics we analysed embryos from mothers expressing 1 copy of Ubq-GFP-Cnn in a *cnn*^{f04547}/*cnn*^{HK21} hemizygous mutant background. For analyzing Cnn band shifts in brain extracts, we used brains from WT or *Sas4*^{S2214} homozygous mutant flies. For analysing the behaviour of WT vs mutant GFP-Cnn constructs, mRNA was injected into embryos or eggs from *cnn*^{f04547}/*cnn*^{HK21} hemizygous mutant mothers. For comparing the effect of GFP-Cnn-WT or GFP-Cnn-10A on the centrosomal localisation of RFP-DSpd-2, we injected the appropriate mRNA into embryos from mothers expressing 1 copy of Ubq-RFP-DSpd-2 in a *cnn*^{f04547}/*cnn*^{HK21} hemizygous mutant background. For analysing MTs in eggs containing GFP-Cnn10D/E aggregates, GFP-Cnn10D/E mRNA was injected into embryos from mothers expressing 2 copies of Jupiter-mCherry.

Antibodies

For western blot analysis, we used the following antibodies: affinity purified rabbit anti-Cnn (1:1000) (Lucas and Raff, 2007), mouse monoclonal anti-α-tubulin (1:1000; DM1a, Sigma), mouse monoclonal anti-Actin (1:2000; A3853, Sigma). Secondary antibodies were from GE healthcare: ECL rabbit or mouse IgG, HRP linked (all used at 1:3000).

Production of centrosome and cytosolic fractions and Phosphatase treatment

Whole centrosomes were isolated from embryonic extracts using a modified version of a centrosome isolation protocol (Lehmann et al., 2006). Briefly, in two separate tubes 7.5ml of embryo extract containing 50% sucrose was layered on top of a sucrose cushion consisting of a 1.5ml layer of 55% sucrose and a 1.5ml layer of 70% sucrose. The tubes were spun in a Beckman SW41 Ti Rotor at 27,000 rpm, causing the centrosomes in the extract to move into the 70% sucrose layer. Five 0.5ml "cytosolic" fractions were taken from the very top of each tube, and four 320μl "centrosome" fractions were collected from the 70% sucrose layer by piercing the bottom of the tube with a 19G needle and collecting the drops. For Phosphatase treatment, 10μl of the centrosomal fraction was incubated with 10μl of Alkaline Phosphatase (Roche) in a 100μl reaction containing protease inhibitors for 4.5h at 37°C. To test the specificity of the Phosphatase treatment, this reaction was carried out in the presence of Phosphatase inhibitor cocktails 2 and 3 (Sigma).

Centrosome immunoprecipitation and mass spectrometry

For immunoprecipitation of centrosomal or cytosolic Cnn molecules 63.9μg of rabbit anti-Cnn antibodies were coupled to 1.25ml protein A conjugated magnetic Dynabeads (Life Technologies). The dynabead/antibody suspensions were rotated at 4°C overnight. The antibodies were cross-linked to the beads using the BS^3 crosslinker (Thermo Fisher Scientific). 2.5ml of the centrosomal and cytoplasmic fractions (obtained as described above) were diluted 1:1 with dilution buffer (PBS with 0.2% BSA, protease inhibitor cocktail, 1mM PMSF, 20μg/ml DNAse I and Phosphatase inhibitor cocktail 2 and 3 (Sigma)) and added to the antibody-beads and rotated at 4°C for 2 h. Beads were washed 3x with wash buffer (PBS with 0.003% Triton X-100, protease inhibitor cocktail, 1mM PMSF, 20μg/ml DNAse I and Phosphatase inhibitor cocktail 2 and 3) and then boiled in 30μl 2xSB. A magnet was used to separate the beads from the sample, and the sample was then separated on a polyacrylamide gel. The band containing Cnn was cut out and stored at -20°C.

Gel pieces were washed with Solution B (100mM ammonium bicarbonate in 50% acetonitrile) for 1-2 h and then washed in 100% acetonitrile for 10 min, before they were dehydrated in a vacuum concentrator (SpeedVac). 10mM DTT was added for 30 min at 37°C and then the gel pieces were washed several times in 25mM ammonium bicarbonate, followed by acetonitrile. Gel pieces were then treated with 55mM iodoacetamide for 1 h in the dark, and subsequently washed several times in Solution B and then in acetonitrile. Gel

pieces were dried in a vacuum concentrator and incubated over night with Promega Sequencing Grade Modified Trypsin (reconstituted and diluted according to manufacturer's instructions). 1μl of formic acid was then added to stop the digest. Supernatant containing the peptides was transferred to a new tube and the gel pieces were incubated with extraction buffer (0.001% formic acid in 50% acetonitrile) for 30 min to extract any remaining peptides, and the supernatant was pooled with the existing supernatant. Samples were dehydrated using a vacuum concentrator.

To enrich for phosphopeptides, dried samples containing tryptic peptides were dissolved in GA solution (80mg/ml glycolic acid in 80% acetonitrile and 2% trifluoroacetic acid). Micro-columns were prepared by plugging the bottom of a GelLoader tip (Eppendorf) with a piece of C8-silica matrix (Varian GmbH) and loading with 5μl TiO_2 slurry (Hichrom Ltd.) followed by a brief centrifugation. The micro-column was washed with 0.6% NH_4OH solution followed by washing with GA solution; the centrifugation speed was adjusted so that the solutions passed through the columns relatively slowly. The dissolved peptides were then loaded on the prepared micro-columns and spun slowly to give phosphopeptides time to bind to the TiO_2. Columns were washed with GA solution followed by 80% acetonitrile + 0.2% trifluoroacetic acid followed by 20% acetonitrile. The bound phosphopeptides were eluted with 0.6% NH_4OH followed with 60% acetonitrile into a new LoBind Eppendorf tube (centrifugation speeds were adjusted to low speeds as phosphopeptides elute

very slowly). Samples were then dried in a vacuum concentrator and dissolved in 5% formic acid for mass spectrometry analysis.

Liquid chromatography-tandem mass spectrometry (LC-MS/MS) analysis was performed using a LTQ Orbitrap Mass Spectrometer (Thermo Scientific) coupled to an UltiMate 3000 Nano LC system (Thermo Scientific).

Production of Cnn mutant constructs

Point mutations were introduced into the *cnn* coding sequence by either QuickChange (Agilent Technologies) or by gene synthesis (Genewiz). For phospho-mutant constructs, serines or threonines were mutated to alanines. For phospho-mimetic constructs, serines were mutated to aspartic acids and threonines to glutamic acids. For LZ mutants, all residues in positions a and d, including six leucines and one lysine, were mutated to alanines.

Protein expression and purification

DNA fragments of *Drosophila* Cnn were cloned into pMAL-C2X vector containing an N-terminal MBP tag. Proteins were expressed in *Escherichia coli* (*E. coli*) Tuner strains and induced with 1mM isopropyl beta-D-1-thiogalactopyranoside (IPTG) at 21°C overnight. Cell pellets were resuspended in lysis buffer (50mM Tris-HCl pH7.5, 150mM NaCl, 3mM b-mercaptoethanol) supplemented with protease inhibitor cocktail (Roche),

DNase I (Sigma) and lysozyme (Sigma). The cells were lysed using an Emulsiflex C5 homogenizer (Avestin) and the insoluble fraction was separated by centrifugation at 19,000 rpm for 30 min. The supernatant was loaded onto the MBP affinity column (GE Healthcare), and the tagged protein was eluted with 10mM maltose in lysis buffer. The protein was further purified by HiLoad-superdex chromatography (HiLoad^{TM} 16/60 Superdex^{TM} 200 prep grade, GE Healthcare).

In vitro Kinase assay

Phosphorylation of purified MBP fusion proteins by Plk1 kinase (ProQinase) was performed in 50mM Tris-HCl pH7.3, 50mM KCl, 10mM $MgCl_2.6H_2O$, 20mM sodium b-glycerophosphate, 15mM EGTA, 1mM DTT, 0.1mM cold ATP, and 1mCi of [γ- ^{32}P]-ATP (PerkinElmer) in a reaction volume of 20μl. The reaction was incubated at 30°C for 30min, and stopped by the addition of 5xSDS sample buffer. The proteins were denatured at 95 ^{o}C for 5min. For SDS PAGE analysis, proteins were separated on the 10% Tris-Glycine gel and stained with Coomassie Brilliant Blue R-250. The gel was then subjected to autoradiography.

Size exclusion chromatography - multi angle light scattering (SEC-MALS)

Proteins were separated using a Superose 6 10/30 GL column (GE healthcare), and the light scattering properties of each sample was detected with a Dawn Heleos-II light scattering detector and an Optilab rEX refractive

index monitor. The running buffer contained 50mM Tris-HCl pH7.5, 150mM NaCl, 5% glycerol and 5mM b-mercaptoethanol. Each run contained 110 μl of protein sample. Molecular mass calculations were performed using ASTRA 6.1.1.17 (Wyatt Technologies) assuming a dn/dc value of 0.186mL/g.

RNA injections

The WT Cnn coding sequences was amplified by PCR and cloned into a pRNA-EGFP-NT vector (Oliveira et al., 2010) using Spe1 and Not1 restriction sites to create a pRNA-EGFP-Cnn construct. PReM domain mutants produced in a pDONR-Zeo-Cnn vector were sub-cloned into the pRNA-EGFP-Cnn construct using Sph1 sites that flank the PReM domain. Capped mRNA was produced by *in vitro* transcription using T3 mMESSAGE mMACHINE (Ambion) and purified using RNeasy MinElute (Qiagen). The mRNA concentration was adjusted to 2mg/ml and injected into either 0-30 min old embryos or eggs from cnn^{f04547}/cnn^{HK21} hemizygous mutant mothers. Embryos were filmed ~60-120 min later, during the syncytial nuclear divisions that occur at the cell cortex, and eggs were filmed ~60-180 min later.

Yeast two-hybrid analysis

Fragments of cDNA to be tested were amplified using a two-step PCR protocol. Initial amplification was carried out using gene specific primers that contained an AscI site in the forward primer and a NotI site and stop codon in the reverse primer. The products from the primary reaction were used as a

template for a secondary PCR reaction, which added vector-specific homology arms.

Primers used for pPC86-AN prey clones:

pPC86AGR_Fwd-CCCAAAAAAAGAGGGTGGGTCGAGGCGCGCC

pPC86NGR_Rev-TTAGAGCTCGACGTCTTACTTACTTAGCGGCCGCG

Primers used for pPC97-AN bait clones:

pPC97AGR_Fwd-GACAGTTGACTGTATCGTCGAGGTCGAGGCGCGCC

pPC97NGR_Rev-CTTAGAGCTCGACGTCTTACTTACTTAGCGGCCGC as in (Boxem et al., 2008).

To create the bait and prey clones 5 μl purified PCR product was transformed with 100 ng cut pPC86-AN or pPC97-AN DNA. Transformation was into either Y8800 (prey plasmids) or Y8930 (bait plasmids) frozen competent yeast cells (Boxem et al., 2008) that were prepared, and used, according to (Gietz and Schiestl, 2007). Haploid yeast strains containing bait or prey plasmids were mated together to make diploid strains containing both bait and prey plasmids; these strains were screened for potential interactions between the bait and prey fragments by plating on media that selected for transcription at the *GAL1::HIS3* locus and the *GAL-2::ADE2* locus. Colorimetric assays were used to test the transcription activity of the *GAL7::LacZ* locus. Auto-activating baits, and *de novo* auto-activators (where auto-activation occurs only after mating) were identified as described in (Vidalain et al., 2004).

Supplementary References

Boxem, M., Maliga, Z., Klitgord, N., Li, N., Lemmens, I., Mana, M., de Lichtervelde, L., Mul, J.D., van de Peut, D., Devos, M., et al. (2008). A protein domain-based interactome network for C. elegans early embryogenesis. Cell *134*, 534–545.

Gietz, R.D., and Schiestl, R.H. (2007). Frozen competent yeast cells that can be transformed with high efficiency using the LiAc/SS carrier DNA/PEG method. Nat Protoc *2*, 1–4.

Lehmann, V., Müller, H., Lange, B.M.H. (2006). Immunoisolation of centrosomes from *Drosophila melanogaster*. Curr Protoc. 3.17.1-13.

Lucas, E.P., and Raff, J.W. (2007). Maintaining the proper connection between the centrioles and the pericentriolar matrix requires Drosophila Centrosomin. J Cell Biol *178*, 725–732.

Oliveira, R.A., Hamilton, R.S., Pauli, A., Davis, I., and Nasmyth, K. (2010). Cohesin cleavage and Cdk inhibition trigger formation of daughter nuclei. Nat Cell Biol *12*, 185-192.

Vidalain, P.O., Boxem, M., Ge, H., Li, S., and Vidal, M. (2004). Increasing specificity in high-throughput yeast two-hybrid experiments. Methods *32*, 363–370.
