# Supplementary figures and images for: The Centrosome-Specific Phosphorylation of Cnn by Polo/Plk1 Drives Cnn Scaffold Assembly and Centrosome Maturation
Source: Dev Cell. 2014 Mar 31;28(6):659–69. doi: 10.1016/j.devcel.2014.02.013 (PMC3988887; doi:10.1016/j.devcel.2014.02.013)

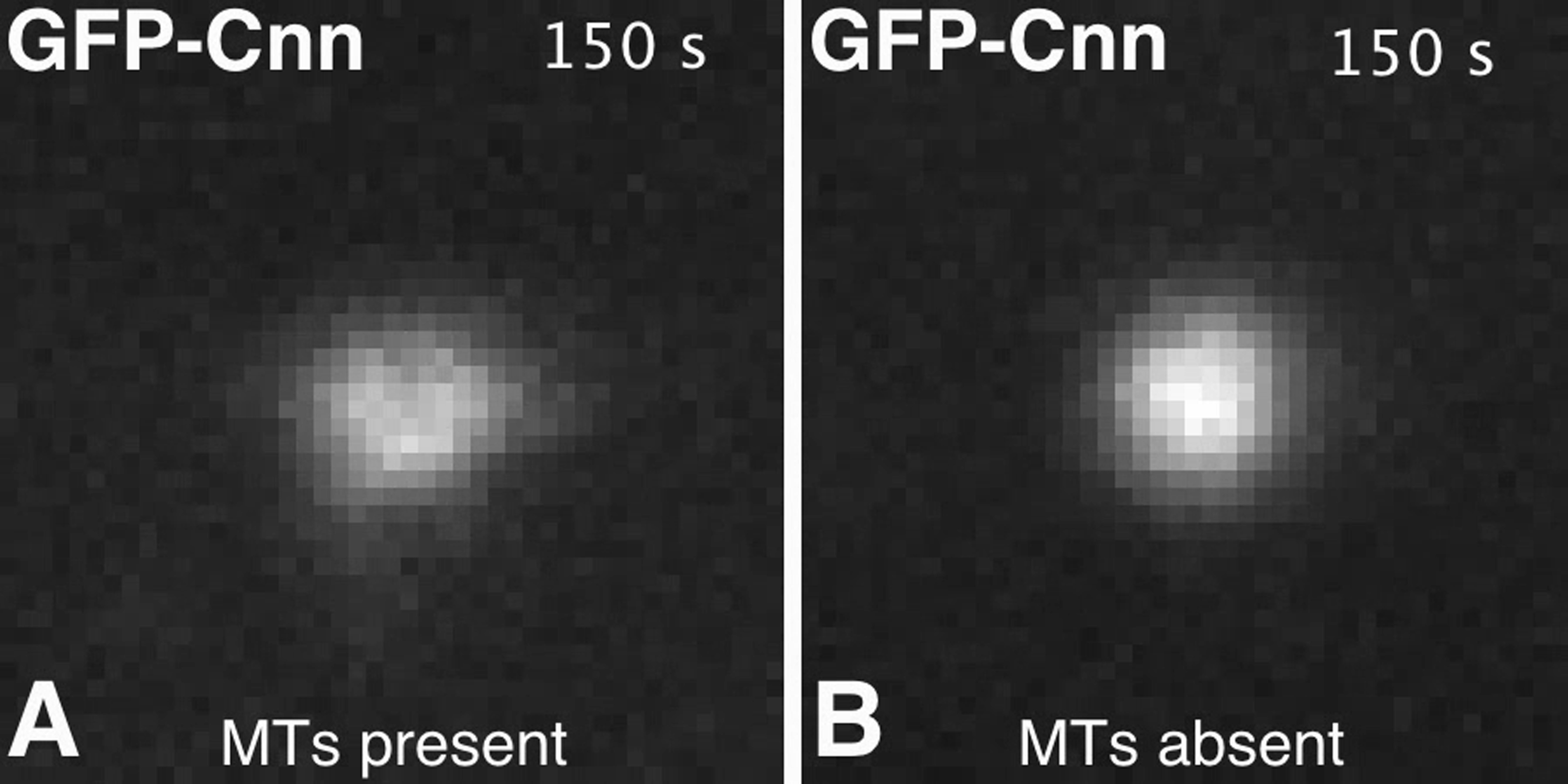

Supplement: Movie S1, Related to Figure 1. GFP-Cnn Molecules Are Initially Incorporated into the Center of the PCM in an MT-Independent Manner — These movies show the results of FRAP experiments illustrating the dynamic behavior of GFP-Cnn at centrosomes in Drosophila embryos with either intact MTs (A) or MTs depolymerized by colchicine injection (B). Time before and after photobleaching at t = 0 is shown at the top right of each panel. Note how, in (A), the GFP-Cnn fluorescence signal initially recovers in the center of the PCM and then spreads outward; in (B), in the absence of MTs, the rate of GFP-Cnn incorporation into the center of the PCM is largely unperturbed, but the outward spread into the periphery is decreased. [file mmc3.jpg]

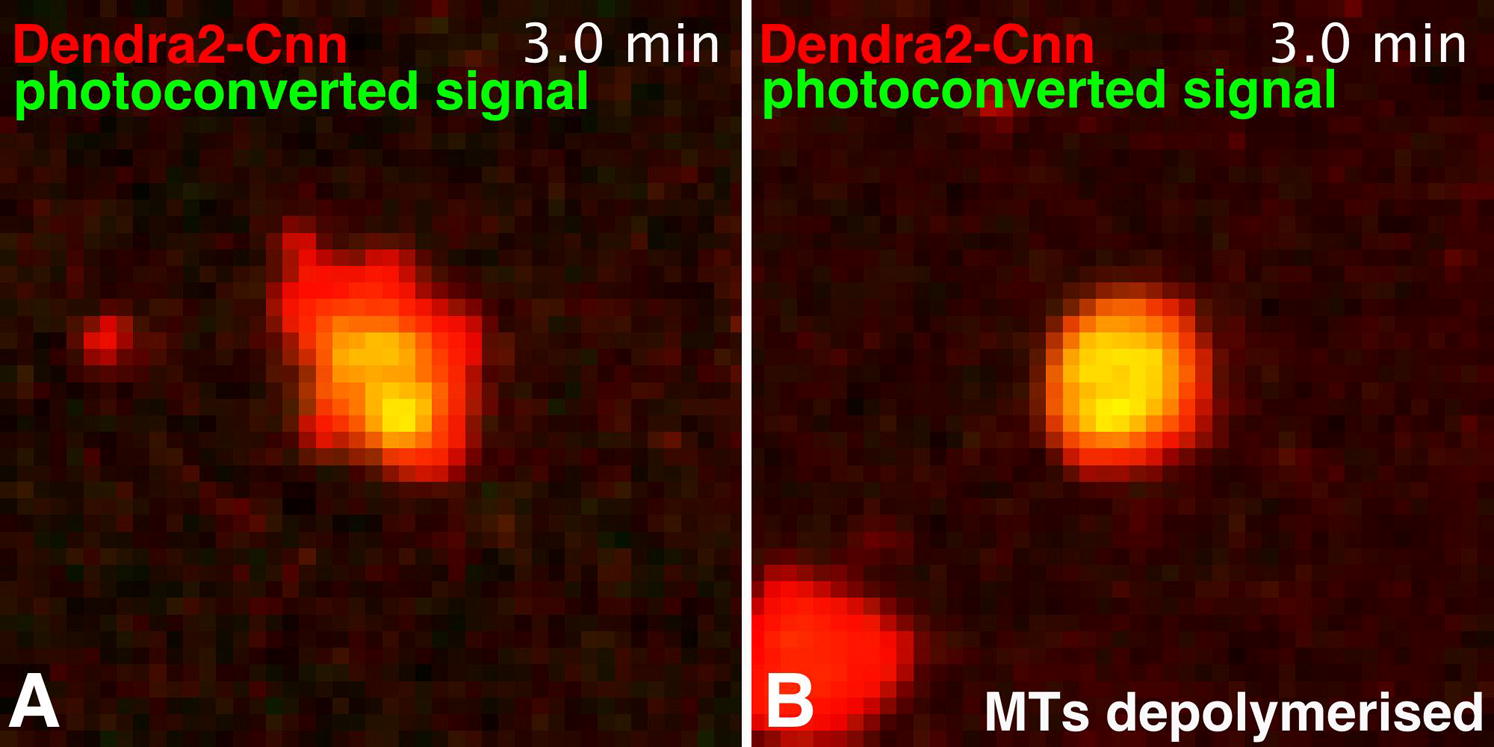

Supplement: Movie S2, Related to Figure 1. GFP-Cnn Molecules Spread Outward through the PCM in a Largely MT-Dependent Manner — These movies show the results of photoconversion experiments illustrating the dynamic behavior of Dendra2-Cnn (pseudocolored red) at centrosomes in Drosophila embryos with either intact MTs (A) or MTs depolymerized by colchicine injection (B). Time from when the molecules in the central region of the centrosome had been photoconverted (pseudocolored green) is shown at the top right of each panel. In each case, photoconversion was carried out in S phase, and the centrosomes were filmed through M phase. When MTs are intact (A), the photoconverted signal gradually spreads outward through the PCM during S phase (t = 0 to t = 2.5). During M phase (t = 3 to t = 6), the movement slows down—Cnn flaring is known to be suppressed during mitosis (ref)—but, when the embryo enters the following S phase (t = 6.5), flaring resumes and the photoconverted molecules rapidly spread outward and are lost from the PCM as flares. This interpretation is consistent with the behavior of photoconverted molecules after MTs have been depolymerized (B), in which the photoconverted molecules initially move outward a short distance (explaining the presence of the dark hollow that becomes weakly visible at the center of the photoconverted molecules) but then remain clustered in the center of the PCM. [file mmc4.jpg]

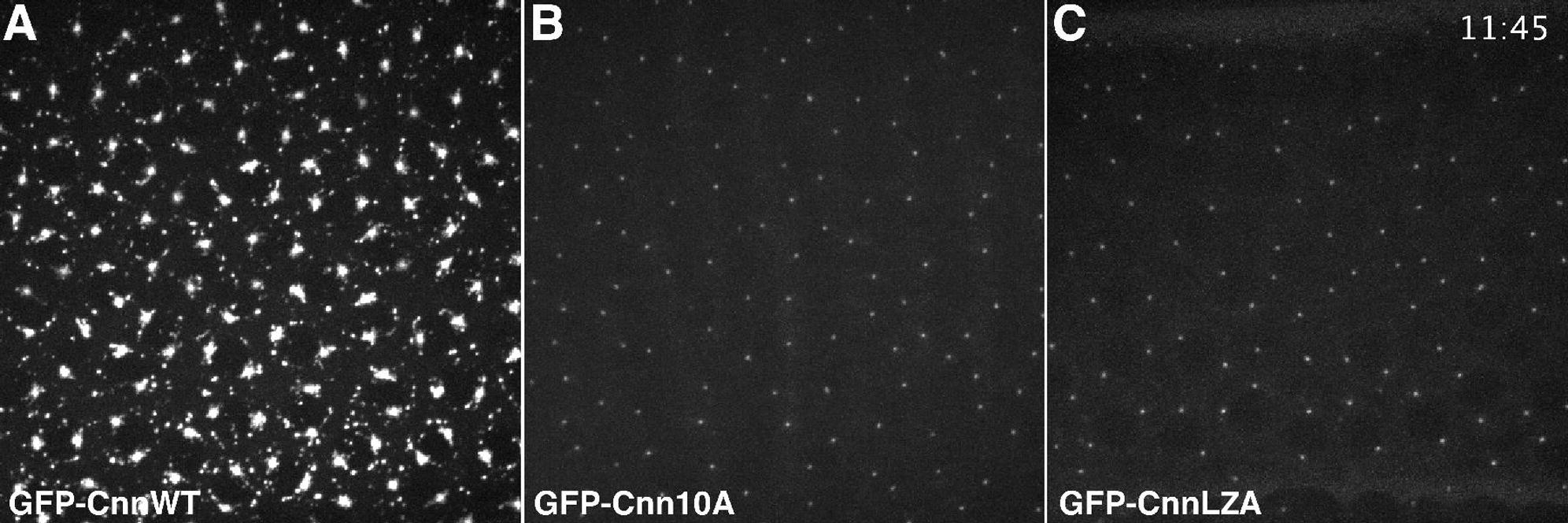

Supplement: Movie S3, Related to Figure 3. Cnn Scaffold Formation Is Dependent on Phosphorylation of the PReM Domain and the Presence of an Intact LZ — These movies show the localization of GFP-CnnWT (A), GFP-Cnn-10A (B), and GFP-Cnn-LZA (C) at centrosomes in cnn null mutant embryos injected with mRNA encoding the respective protein. Time after the start of each movie is shown at the top right of (C). (A) GFP-Cnn WT exhibits a similar localization to that seen with WT GFP-Cnn provided transgenically (see Figure 1A; Movie 1A; data not shown), and the protein appears to efficiently rescue the cnn null mutant embryo phenotype (the nuclei and spindles within embryos laid by cnn mutant mothers are normally severely misorganized). (B and C) When either the PReM domain phosphorylation sites (B) or the a and d positions within the PReM domain LZ (C) are mutated to alanine, however, the amount of GFP-Cnn at centrosomes is reduced and GFP-Cnn displays a much tighter distribution around the centrioles. The cnn null mutant phenotype is at least partially rescued, because the nuclei and spindles are better organized than those of cnn null mutant embryos. [file mmc5.jpg]

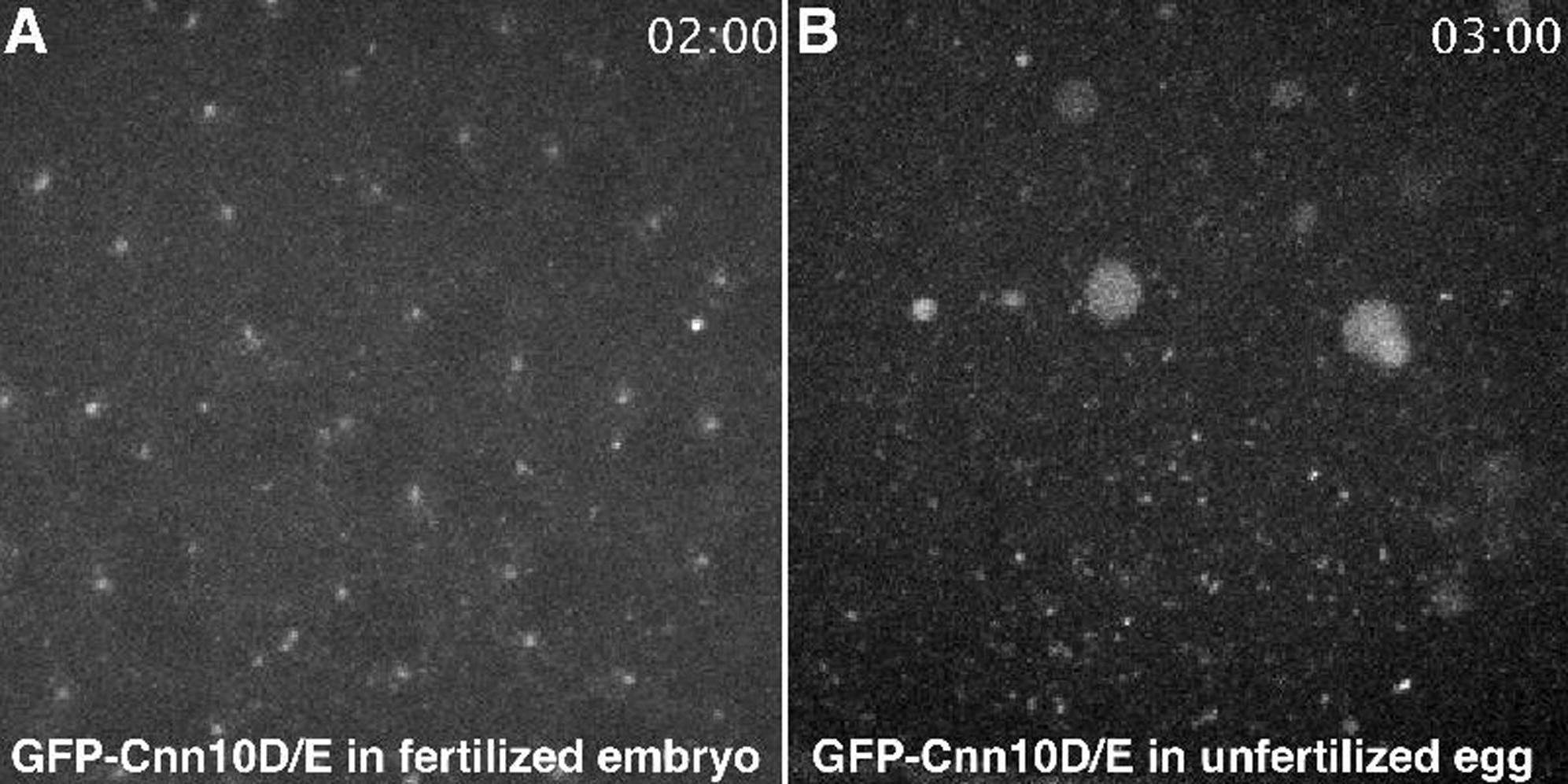

Supplement: Movie S4, Related to Figure 4. GFP-Cnn Foci Form Spontaneously in the Cytosol When Phosphorylation within the PReM Domain Is Mimicked by Mutation — These movies show the behavior of GFP-Cnn-10D/E in cnn null mutant Drosophila embryos (A) or unfertilized eggs (B) injected with mRNA encoding GFP-Cnn-10D/E. Time since the start of each movie is shown at the top right of each panel. (A) In embryos, GFP-Cnn-10D/E localizes to centrosomes but also forms independent cytosolic foci that increase in size over time. The centrosomes can be seen clearly at the beginning of the movie but become increasingly difficult to distinguish as the movie progresses because of buildup of the cytosolic GFP-Cnn-10D/E foci. (B) GFP-Cnn-10D/E foci also form in unfertilized eggs, which lack centrosomes, showing that the foci form spontaneously in the cytosol independently of centrosomes. These foci also increase in size over time. The larger (but more weakly fluorescent) round objects in the unfertilized egg (B) are yolk granules that autofluoresce. [file mmc6.jpg]

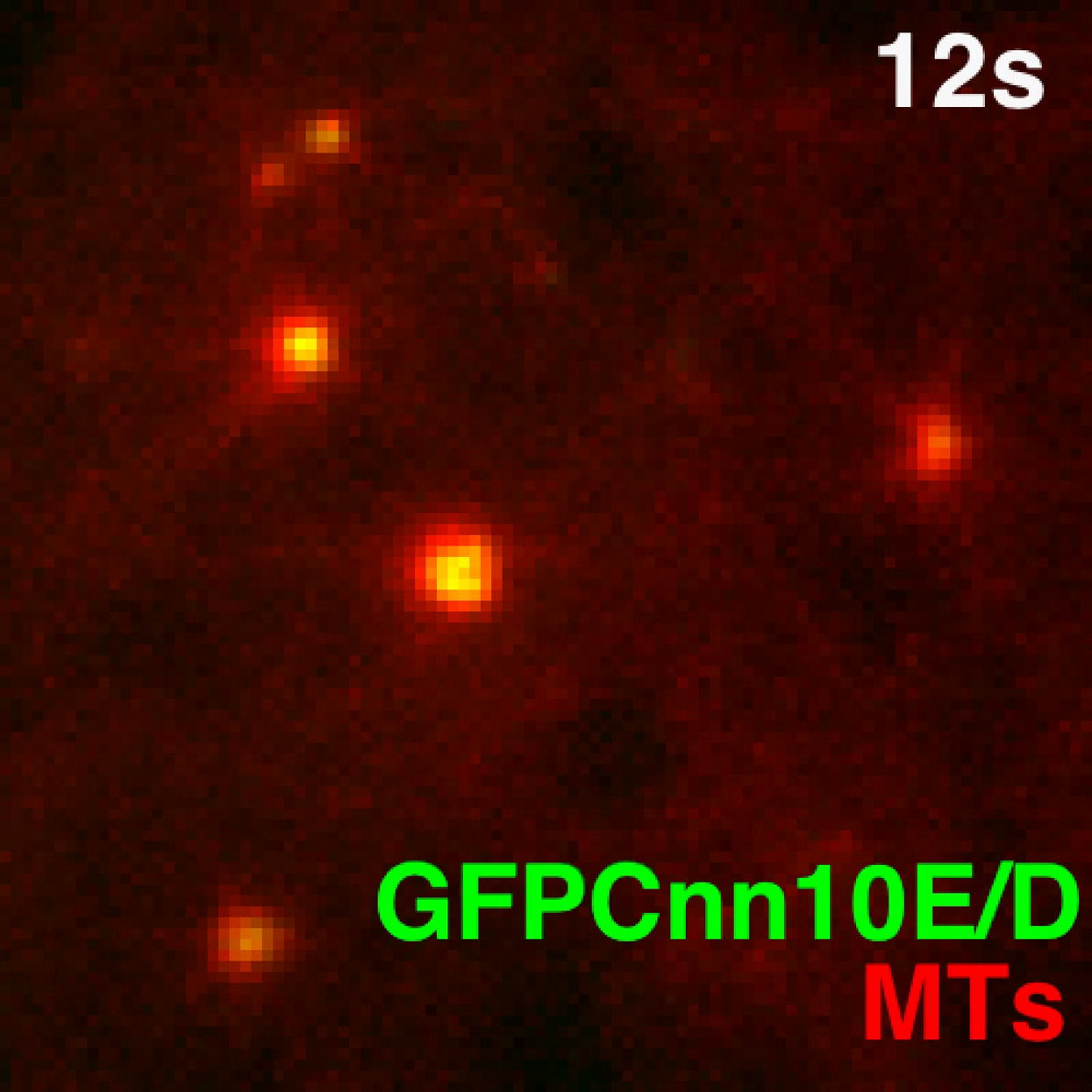

Supplement: Movie S5, Related to Figure 4. GFP-Cnn10E/D Foci that Form Spontaneously in the Cytosol Can Organize Dynamic MTs — This movie shows GFP-Cnn10D/E foci (green) in unfertilized eggs expressing the MT marker Jupiter-mCherry (red). Time since the start of the movie is shown at the top right. At this resolution, individual MTs are impossible to discern, but the MTs organized by the GFP-Cnn10D/E foci appear to be dynamic. [file mmc7.jpg]
